# Integrating single-cell RNA and bulk RNA sequencing data to identify prognostic genes associated with pyrimidine metabolism in triple-negative breast cancer by machine learning algorithm combinations

**DOI:** 10.1007/s12672-026-05118-6

**Published:** 2026-04-30

**Authors:** Xue Gong, Yubo Du, Huanyu Zhang, Keru Ma, Dongxu Yang, Tianyu Chen, Sunbin Dai, Dalin Li

**Affiliations:** Department of Breast Surgery, Harbin Medical University Cancer Hospital, Harbin, 150086 People’s Republic of China

**Keywords:** Triple-negative breast cancer, Pyrimidine metabolism, 101 machine learning algorithm combinations, Epithelial cells, Prognostic genes

## Abstract

**Background:**

Pyrimidine metabolism plays a crucial role in DNA synthesis and cell proliferation and is associated with the development of various cancers. However, their prognostic value in triple-negative breast cancer remains to be further investigated. This study aimed to identify the prognostic genes related to pyrimidine metabolism in triple-negative breast cancer (TNBC).

**Methods:**

All data applied in this study were obtained from public databases. The prognostic genes related to pyrimidine metabolism in TNBC were identified through Weighted gene co-expression network analysis (WGCNA), differential expression analysis, univariate Cox regression, and 101 combinations of machine learning algorithms. Based on the optimal model among the 101 combinations of machine learning algorithms, a risk model was built. Then, the risk model was validated in GSE58812 dataset. Subsequently, a nomogram was built based on prognostic genes. Meanwhile, enrichment analysis, gene mutation analysis, and drug sensitivity analysis were also executed. In addition, the somatic mutation analysis of TNBC patients revealed that missense mutations were the predominant mutation type. To explore the cells that influence the risk of TNBC, single-cell RNA sequencing analysis was conducted. Finally, the expression levels of prognostic genes were verified through qPCR experiments. SEPT3 expression in TNBC cells was detected by qPCR; its effects on TNBC cell proliferation (CCK-8) and invasion/migration (Transwell, wound-healing) were evaluated.

**Results:**

Firstly, 5 prognostic genes (ECE2, NFE2L3, PFKFB3, FADS2, and SEPT3) were identified. The risk model demonstrated high reliability, and the survival rate of high-risk patients was relatively low. A nomogram with relatively high prognostic accuracy was constructed. Next, 22 pathways were identified by enrichment analysis. A drug sensitivity analysis identified 12 drugs were identified (e.g. ABT.263). In addition, epithelial cells were related to the expression of the prognostic genes.

**Conclusion:**

ECE2, NFE2L3, PFKFB3, FADS2, and SEPT3 are associated with pyrimidine metabolism in TNBC. A risk model and nomogram were successfully constructed based on these genes, providing a theoretical basis for the treatment of TNBC patients. We confirm the model’s validity in TNBC by validating SEPT3 (a key PyMRG) regulates TNBC cell proliferation, migration, and invasion.

**Supplementary Information:**

The online version contains supplementary material available at 10.1007/s12672-026-05118-6.

## Background

 Breast cancer (BC) ranks among the most prevalent malignancies affecting women globally [1]. Among its subtypes, triple-negative breast cancer (TNBC) is recognized as one of the most aggressive forms, which is linked to an unfavorable prognosis. TNBC is a specific type of breast cancer characterized by the absence of estrogen receptors (ER), progesterone receptors (PR), and human epidermal growth factor receptor 2 (HER2) [2]. TNBC accounts for approximately 15% of all breast cancer cases, and due to the lack of endocrine and anti-HER2 therapeutic targets, there is a lack of effective targeted therapies and no established standard treatment regimen; furthermore, the underlying molecular mechanisms of TNBC have yet to be fully clarified. Studies have revealed that TNBC involves various molecular alterations, including genetic mutations and dysregulated signaling pathways [3, 4]. Identifying novel prognostic biomarkers for TNBC is crucial for advancing our understanding of its molecular mechanisms, discovering new therapeutic targets, and improving clinical outcomes.

Pyrimidine metabolism refers to a set of biochemical reaction processes in living organisms for the synthesis and degradation of pyrimidine-type compounds [5, 6]. Pyrimidine metabolism (PyM) comprises three interrelated pathways: (1) salvage of free bases and nucleosides, (2) de novo synthesis that is founded on ribose-based precursors and amino acid substances, and (3) catabolism of excess nucleosides and nucleotides [7].

Studies of pyrimidine metabolism in various cancers suggest that abnormalities in the pyrimidine biosynthesis pathway may be closely linked to tumor initiation and progression [8, 9]. Accumulating studies have established that key enzymes in the de novo pyrimidine biosynthesis pathway—such as dihydroorotate dehydrogenase (DHODH)—sustain the hyperproliferative phenotype of cancer cells and suppress ferroptosis through remodeling mitochondrial redox homeostasis, thereby driving tumor progression [10, 11]. Notably, dihydropyrimidine dehydrogenase (DPD), a central enzyme in pyrimidine catabolism, directly modulates the metabolic fate and therapeutic potency of the chemotherapeutic agent 5-fluorouracil(5-FU) [12]. 5-FU is a first-line chemotherapeutic for BC and other malignancies; yet, ~ 80% of administered 5-FU is catabolized by DPD into inactive dihydrofluorouracil, a process that markedly diminishes its antitumor activity [13, 14]. Accordingly, intratumoral DPD expression levels have been widely validated as both predictive and prognostic biomarkers for patients receiving 5-FU-based chemotherapy [12, 15]. Furthermore, in BC, dysregulation of pyrimidine metabolism correlates with enhanced tumor cell proliferation. Specifically, key enzymes in pyrimidine metabolism—including thymidylate synthase (TS)—support the maintenance of a dedifferentiated phenotype in TNBC cells, ultimately accelerating tumor progression [8]. In light of these findings, targeting PyM may be an attractive approach for the treatment of cancer, including TNBC. Currently, further investigations are needed to explore the prognostic role and molecular mechanisms of pyrimidine metabolism-related genes in TNBC.

Understanding the functions of these genes may provide new insights for early diagnosis and targeted therapy of TNBC. The application of machine learning in disease-related gene research mostly involves the integration of multi-omics data (such as gene expression and proteomics) and the use of algorithms to identify key genes and biomarkers. This process helps to support disease diagnosis, prognosis and treatment [16–18]. In cancer research, 101 combinations of machine learning algorithms are widely used to build prognostic models and identify potential therapeutic targets [19]. For example, by combining ten common machine learning algorithms, researchers have been able to identify genes associated with cancer prognosis from transcriptomic data, and construct highly accurate prediction models [20]. However, despite the use of various algorithms in cancer research, the prognostic value and molecular mechanisms of these approaches in specific cancers, such as triple-negative breast cancer, require further investigation.

single-cell RNA sequencing (scRNA-seq) technology analyzes data at the single-cell level, which can clearly present the distribution and functional states of different cell types within tissues, and greatly improve the resolution and accuracy of the data [21]. In particular, it is of great significance in the field of cancer research, where as it can elucidate the heterogeneity of tumors, dynamic changes in the tumor microenvironment and the evolution of cancer cells, and open up new perspectives for cancer diagnosis and drug resistance research [22]. From a clinical point of view, it helps to deeply analyze the molecular mechanisms of diseases and build a solid foundation for the innovation of prevention, diagnosis, and treatment strategies, and combined with transcriptome sequencing, it facilitates the identification of key disease-related genes in multiple dimensions [23].

In this research endeavor, transcriptomic data sourced from publicly accessible databases were employed. Employing a comprehensive approach that incorporated weighted gene co-expression network analysis (WGCNA), differential expression analysis, univariate Cox regression analysis, and an intricate combination of 101 machine learning algorithms, the study aimed to pinpoint genes associated with the prognosis of TNBC and subsequently construct a prognostic model. Subsequently, building upon the genes that were identified as being related to prognosis, a nomogram was meticulously developed. To further delve into the molecular mechanisms, gene set variation analysis (GSVA) enrichment analysis was conducted, with the specific objective of exploring and discerning the pathway discrepancies between the high-risk and low-risk groups. Single-cell datasets were then used to investigate cells associated with prognostic gene expression. Drug sensitivity analysis helped to identify potential therapeutic drugs for TNBC. Finally, the expression levels of prognostic genes were verified through qPCR experiments. This study provides a theoretical basis for improving the prognosis of TNBC patients and promises to improve treatment strategies for this group of patients.

## Methods

### Data collection

The gene expression profiles and survival information of the Cancer Genome Atlas (TCGA)-TNBC were acquired from University of California, Santa Cruz (UCSC) Xena database (https://xena.ucsc.edu/) (access time: September 19, 2024). The TCGA-TNBC dataset comprised 121 TNBC samples with complete survival information and 113 normal tissue samples. The GSE58812 dataset (GPL570) was subsequently acquired from the Gene Expression Omnibus (GEO) database (https://www.ncbi.nlm.nih.gov/geo/), consisting of 107 tumor tissue samples with complete survival information. In addition, the single-cell transcriptome dataset for TNBC, GSE176078 (*n* = 9), was also acquired from GEO database. At the same time, the 105 pyrimidine metabolism related genes (PyMRGs) were downloaded from the published literature [24] (Table S1).

### WGCNA

In TCGA-TNBC dataset, PyMRGs were successively subjected to univariate Cox analysis (HR ≠ 1 and *P* < 0.2) and PH assumption test (*P* > 0.05). The genes that passed PH assumption test were used as the basis, and ssGSEA scores for each TNBC samples in the TCGA-TNBC dataset were calculated through the GSVA package (v 1.50.0) [25]. The disease categories were categorized into high and low scoring categories based on optimal cut-off value of PyMRGs scores. By means of the survminer package (v 0.4.9) [25], the KM survival analysis was performed in high and low scoring groups. The survival disparity between two scoring groups was tested by log-rank test (*P* < 0.05).

Next, the PyMRGs scores were used as a trait for WGCNA with the WGCNA package (v 1.71) [26]. The first step involved the execution of hierarchical clustering on the 121 disease samples. Subsequently, a soft threshold was selected based on the scale-free topology, with R^2^ = 0.9. The construction of the scale-free network was performed in accordance with the soft threshold, in order to obtain the gene co-expression modules. The relationships between modules and PyMRGs scores were calculated by Pearson analysis (|cor| > 0.3 and *P* < 0.05), and correlation heatmaps were constructed. The key module genes were selected from the highest and lowest correlation modules and named PyMet-Genes.

### Differential expression analysis

Next, differentially expressed genes (DEGs) between TNBC and normal samples in TCGA-TNBC dataset were ascertained by the DESeq2 (v 1.38.0) [27], with *P* < 0.05 and |log_2_ Fold Change (FC)| > 0.5. Volcano plot labelling top 10 up- and down-regulated genes (log_2_FC) in DEGs was generated with the ggplot2 package (v 3.5.1) [28]. Heatmap depicting the expression of DEGs was created with pheatmap (v 1.0.12) [29].

### Functional enrichment analysis

VennDiagram (v 1.2.3) [30] was used to create intersections between DEGs and PyMet-Genes. Thereafter, kyoto encyclopedia of genes and genomes (KEGG) and gene ontology (GO) analyses were performed for intersecting genes by clusterProfiler (v 4.10.1) [31] (*P* < 0.05) to investigate the functions of intersecting genes.

### Built and verification of the risk model

In TCGA-TNBC, the prognosis-related genes were ascertained through univariate Cox analyses (HR ≠ 1 and *P* < 0.05) and PH assumption tests (*P* > 0.05) by survival package (v 3.5.3) [32], based on the candidate genes. Subsequently, 101 combinations of machine learning algorithms combinations were fitted based on 10 machine learning algorithms by leave-one-out cross-validation (LOOCV) framework in the TCGA-TNBC dataset and the GSE58812 dataset. The 10 machine learning algorithms included RF (randomForest package (v 3.2.2) [33]), Enet (glmnet package (v 4.1.8) [34]), least absolute shrinkage and selection operator (LASSO) (glmnet package (v 4.1.8)), Ridge (glmnet package (v 4.1.8)), stepwise Cox (survival package (v 3.5.3)), CoxBoost (CoxBoost package (v 1.4) [35]), plsRcox (plsRcox package (v 1.5.1) [36]), SuperPC (superpc package (v 1.12) [37]), GBM (gbm package (v 2.1.9) [38]), and survival-support vector machine (SVM) (survivalsvm package (v 0.0.5) [39]). The C-index for each model was computed within two datasets. Model possessing highest C-index, with a value exceeding 0.6, was chosen as the optimal one. Subsequently, this selected optimal model was utilized to identify the prognostic genes.

Risk scores were calculated for each sample derive from the optimal model. Risk score was calculated by formula as follows: Risk score $\:={\sum\:}_{i=1}^{n}\:{\beta\:}_{i}\ast\:$ gene_expression. The “$\:{\beta\:}_{i}$” is the prognostic gene i coefficients in optimal model, and “gene_expression” indicates the expression of prognostic gene i. Concurrently, the TNBC patients were divided into high-risk group (HRG) and low-risk group (LRG) relied on optimal cutoff value of the risk score, and risk score curves and survival status plots were demonstrated. Subsequently, Kaplan-Meier (K-M) survival curves for HRG and LRG were generated using survival package (v 3.5.3). The disparity in survival status between HRG and LRG was analyzed by Peto-Peto test (*P* < 0.05). The timeROC package (v 0.4) [40] was utilized to construct receiver operating characteristic (ROC) curves for TNBC patients at 1, 3, and 5 years. Validation of the risk model was both performed in the GSE58812 dataset.

### Construction of nomogram

To assess predictive value of prognostic genes in the TNBC, rms package (v 6.5.0) [41] was employed for the nomogram construction based on the prognostic genes. Subsequently, predictive performance of the nomogram was evaluated in TCGA-TNBC by plotting calibration curves and ROC curves (1, 3, and 5 years).

### Enrichment of TFs motifs

The enrichment of transcription factors for prognostic genes was carried out using the RcisTarget package (v 1.18.2) [42]. TF motifs were retrieved based of the prognostic genes. The Normalized Enrichment Score (NES) of motifs was contingent upon the quantity of motifs present in the database. Additionally, the motifs were annotated using annotation files, which were further deduced based on the similarity of motifs and gene sequences. Subsequently, the area under the curve (AUC) values were computed by means of the cumulative recovery curves of the motifs. Finally, transcriptional factors (TFs) of the prognostic genes were identified among all the enriched motifs.

### GSVA

To determine the differences in biological functions between HRG and LRG, a GSVA was conducted using the GSVA package (v 1.50.0) [43]. The background gene set was “c2.cp.kegg.v2022.1.Hs.symbols.gmt” from Molecular Signatures Database (MSigDB) database (https://www.gsea-msigdb.org/gsea/msigdb). First, the background gene set scores of each TNBC sample were calculated by the single-sample gene set enrichment analysis (ssGSEA) function. Next, limma package (v 3.58.1) [43] was utilized to compare the score disparity of background gene sets between HRG and LRG (*P* < 0.05, |t| > 0) to further analyze the biological functions between the groups.

### Somatic mutation analysis

Somatic mutation pertains to the post-natal changes that occur at the cellular level within particular tissues or organs of an individual and holds a crucial position in the process of tumorigenesis. The somatic mutation analysis for TCGA-TNBC patients was carried out by employing the maftools package (v 2.14.0) [43]. Waterfall plots were generated to depict the top 20 mutated genes across different risk groups.

### The scRNA-seq analysis

The Seurat package (v 5.1.0) [44] was invoked for scRNA-seq analysis within GSE176078 dataset. Initially, scRNA-seq data was processed into Seurat objects and underwent stringent quality control measures. The quality control criteria involved retaining cells with nFeature_RNA ranging from 200 to 6000, ensuring that nCount_RNA ranged from 200 to 30,000.

After logarithmic normalization, the FindVariableFeatures function of Seurat (v 5.1.0) was utilized to determine the top 2000 highly variable genes. The result was visualized using the LabelPoints function, and the top 10 genes with the greatest variations were labeled. Subsequently, these genes were subjected to principal component analysis (PCA) by the RunPCA function. The scree plot was generated based on PCs, and the number of PCs corresponding to where the curve starts leveling off was selected. Next, FindNeighbors and FindClusters functions were utilized to identify small cell clusters, and the Uniform manifold approximation and projection (UMAP) algorithm was applied to perform clustering analysis on the selected PCs (resolution = 0.3). The Seurat package (v 5.1.0) along with the FindAllMarkers function was used to identify the DEGs within distinct cell clusters. Afterwards, these DEGs were incorporated with the marker genes documented in the extant literature for the annotation of cell clusters [45]. The expression patterns of marker genes across diverse cell clusters were visualized via bubble plots.

In addition, to explore the biological pathways in which the annotated cells are involved, the analyze_sc_clusters function in the ReactomeGSA package (v 1.12.0) [46] was used to perform a functional enrichment analysis for each cell type (*P* < 0.05). After that, to explore the interactions between the annotated cells, the CellChat package (v 1.6.1) [47] was utilized to carry out cell communication analysis (*P* < 0.05). Finally, an analysis was executed to map out the expression of prognostic genes across different cell types.

### Drug sensitivity analyses and molecular docking

To evaluate the sensitivity of HRG and LRG patients to chemotherapeutic agents, the chemotherapeutic and targeted therapy drugs applicable to TNBC were obtained from the Genomics of Drug Sensitivity in Cancer (GDSC) database (https://www.cancerrxgene.org/). In context of TCGA-TNBC, pRRophetic package (v 0.5) [48] was utilized to predict half-maximal inhibitory concentrations (IC50) values of chemotherapy/targeted therapy drugs for each TNBC patient. Subsequently, Wilcoxon test was conducted to compare the disparities in drug IC50 between HRG and LRG.

Then, in order to gain an in-depth understanding of how the prognostic genes bind to drugs, based on the drugs with differences, for each gene, the drug with the highest Interaction Score was selected for molecular docking. The three-dimensional structures of the proteins of the genes were downloaded from the Protein Data Bank (PDB) (https://www.rcsb.org/) database, and the two-dimensional structures of the molecular ligands (key active ingredients) were obtained from the PubChem database (https://pubchem.ncbi.nlm.nih.gov/). Subsequently, the proteins and ligands were uploaded to the cb-dock2 online website (http://cadd.labshare.cn/cb-dock2/index.php) for molecular docking, and the binding free energy was calculated. A docking binding energy less than − 5 kJ/mol indicated a good binding ability.

### RNA reverse transcription

Use ReverTra Ace qPCR RT Kit (FSQ-101, TOYOBO, JAPAN). Incubate RNA at 65℃ for 5 min, then ice-preserve. Prepare 10 µl system (RNase-free water, 2 µl 5xRT Buffer, 0.5 µl RT Enzyme Mix, 0.5 µl Primer Mix, 0.5pg-1 µg RNA). Run PCR program: 37℃ for 15 min → 98℃ for 5 min → 4℃ (≤ 1 h). Dilute cDNA 5x with RNase-free water, store at -20℃.

### qPCR assay

The expression difference of prognostic genes in TNBC and control categories was analyzed by qPCR test. Human breast tumor cells (MCF-7, MDA-MB-231, BT-549), and human breast cells (MCF 10 A) were purchased from Procell Life Science & Technology Co., Ltd. (Wuhan, China). MCF 10 A/MCF-7/BT-549/MDA-MB-231 were cultured in RPMI 1640 medium supplemented with 10% fetal bovine serum. qPCR reactions were performed using SYBR Green Realtime PCR Master Mix (Thermo Fisher Scientific, USA) on a quantitative PCR instrument (ABI 7500, Thermo Fisher Scientific, USA). ACTB was selected as the reference gene due to its stable expression across all tested cell lines, and all primer sequences are listed in Table S6. Each reaction was run in 3 technical replicates. Ct values ranged from 15 to 35. Primers: 20–30 mer, 40%-60% GC, target < 200 bp. Use autoclaved EP tubes, operate on ice. Prepare 10 µl system (3 µl RNase-free water, 5 µl mix (1x), 0.5 µl Primer F/R (10µM, 0.4µM final), 1 µl cDNA). Run cycles: pre-denaturation (95℃, 30s) → 40 cycles (95℃ 5s, 65℃ 10s, 72℃ 15s)→melting curve. Retain single-peak data; calculate: ΔCT = CT(target)-CT(internal ref), ΔΔCT = ΔCT(experimental)-ΔCT(control), target expression = 2^-ΔΔCT^.

### Cell transfection

Transfection was performed when the cell confluency reached 80–90%. First, 3 × 10⁵ cells were seeded per well in 6-well plates. Subsequently, using Lipofectamine 3000(L3000001; Invitrogen, USA)reagent and vectors, the negative control (NC), target plasmid (pGLV-CMV-MCS-EF1-copGFP-Puro-SEPT3), and target siRNA (SEPT3 siRNA) were sequentially transfected into BT-549 and MDA-MB-231 cells. The expression level of SEPT3 was detected by qPCR to evaluate the cell transfection efficiency. All primer sequences are listed in Table S6.

### CCK-8 assay

Evaluate MDA-MB-231/BT-549 proliferation after SEPT3 OE/SH. Digest cells, resuspend in complete medium, count. Adjust density to 3000–5000 cells/well (96-well plate). Culture 24,48 h,72 h,96 h replace with CCK-8 solution (10 µl reagent + 90 µl medium/well). Incubate 2–4 h, measure OD at 450 nm, analyze data.

### Wound healing assay

Draw equally spaced lines on 6-well plate bottom. Seed cells, culture with complete medium. When confluent, scratch vertically with 200 µl pipette tip. Wash 2x with PBS, replace with serum-free medium. Record scratch initial state, take photos at set time points, analyze with ImageJ. Microscopic images were acquired using an MIF2-UBY inverted microscope (MingMei, China) equipped with a MD×10 digital camera.

### Transwell invasion assay

Thaw Matrigel on ice, dilute 1:3 with serum-free medium. Add 40 µl to Transwell upper chamber, incubate 2 h at 37℃ to solidify. Digest, centrifuge, count good cells. Resuspend cells in 200 µl serum-free medium, add to upper chamber; add 700 µl complete medium to 24-well lower chamber. Culture, aspirate medium, wash 2x with PBS. Fix with 4% paraformaldehyde (30 min, RT), wash 2x. Stain with 0.1% crystal violet 10 min, wash. Air-dry, take photos, analyze with ImageJ.

### Statistical analysis

Bioinformatics analyses were executed by R (v 4.2.2). Wilcoxon test was invoked to compare the disparity between two groups (*P* < 0.05). qPCR Data were represented as the mean ± standard deviation (SD) and analyzed by GraphPad Prism 9.5.0. The Shapiro-Wilk and Levene’s methods were used to test whether the relevant data conformed to normal distribution and satisfied the homogeneity of variance. When the data conformed to normal distribution and the variance was homogeneous, one-way analysis of variance (ANOVA) was used for multiple group comparisons; when the variance was heterogeneous, non-parametric tests were adopted. In this study, the qPCR data conformed to normal distribution, and the t-test was used to compare the differences between each group of BC cell lines and breast cells, *  P< 0.05, **  P< 0.01, ***  P< 0.001, and **** *P* < 0.0001 were considered as different statistical significances. “ns” stood for no significant difference.

## Results

### The 366 candidate genes were ascertained

**Fig. 1:**
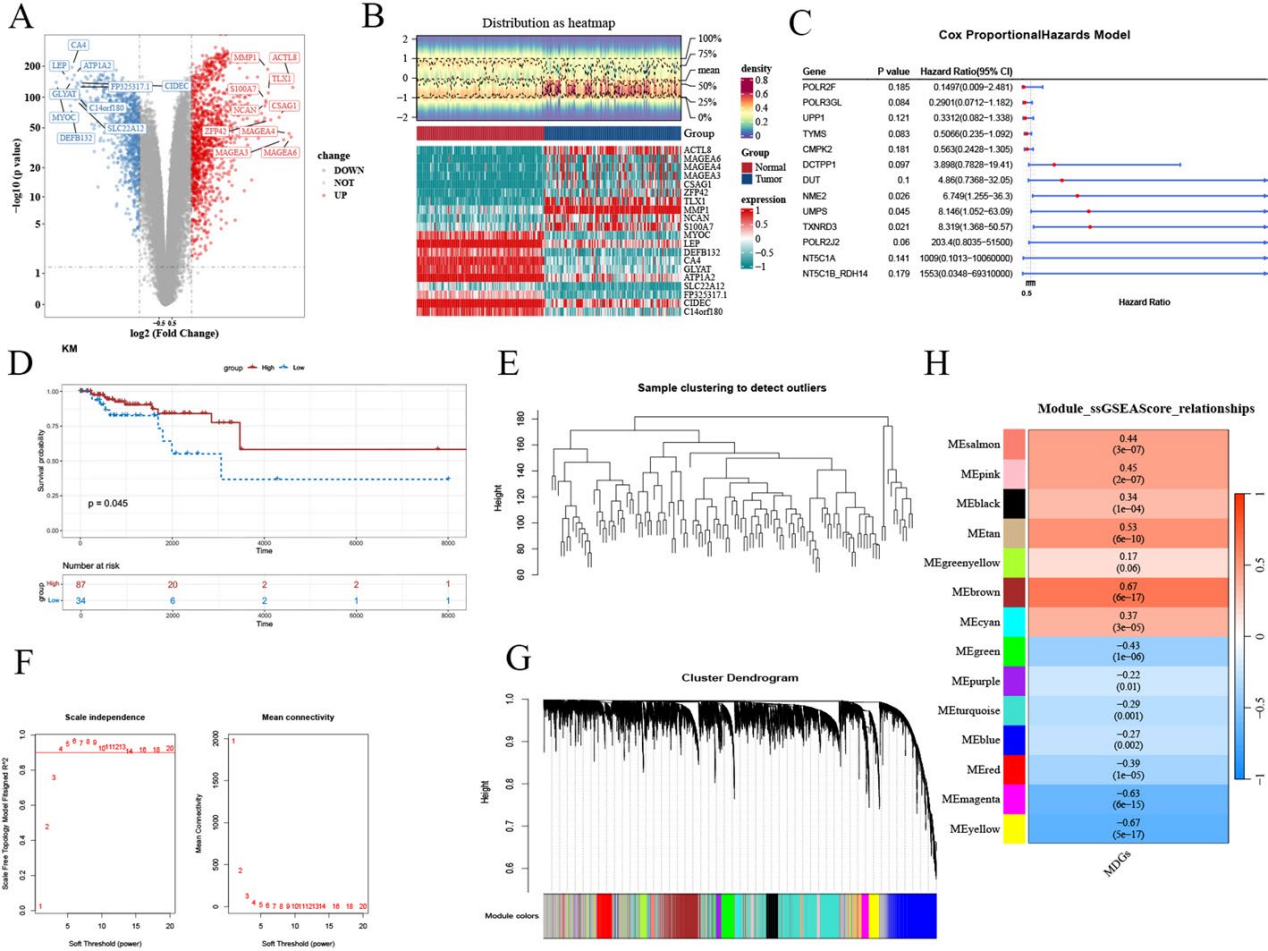
Identification of candidate genes in TNBC. **A** Volcano plot of DEGs. **B** Heatmap of DEGs. The upper part is a density heatmap of the expression levels of differentially expressed genes. The lower part is an expression heatmap. **C** 11 genes passed the univariate cox. **D** KM survival analysis of the high and low score groups. **E** Sample clustering. **F** Soft threshold screening. **G** Module clustering diagram. **H** Heatmap of the correlation between modules and ssGSEA score of PyMRGs

A total of 2210 DEGs were identified between TNBC and control groups in TCGA-TNBC dataset. Among these, 1289 DEGs showed up-regulation while 921 DEGs displayed down-regulation patterns in TNBC group. The top 10 up- and down-regulated genes were labelled in volcano plot (Fig. 1A). A heatmap was generated to show the expression of the DEGs (Fig. 1B).

**Table 1:** Univariate Cox regression and PH assumption test results for candidate pyrimidine metabolism‑related genes

	*p*.value (univariate cox)	HR (95% CI for HR)	*P *(PH)
NT5C1A	0.141	1009 (0.1013-10060000)	0.231314
CMPK2	0.181	0.563 (0.2428–1.305)	0.42594
NT5C1B_RDH14	0.179	1553 (0.03479–69310000)	0.701939
UMPS	0.0446	8.146 (1.052–63.09)	0.07412
UPP1	0.121	0.3312 (0.08199-1.338)	0.188892
POLR2J2	0.0598	203.4 (0.8035–51500)	0.712388
DUT	0.1	4.86 (0.7368–32.05)	0.334685
DCTPP1	0.0967	3.898 (0.7828–19.41)	0.287559
NME2	0.0261	6.749 (1.255–36.3)	0.712702
TYMS	0.0827	0.5066 (0.235–1.092)	0.308673
POLR2F	0.185	0.1497 (0.009029-2.481)	0.358409

**Fig. 2:**
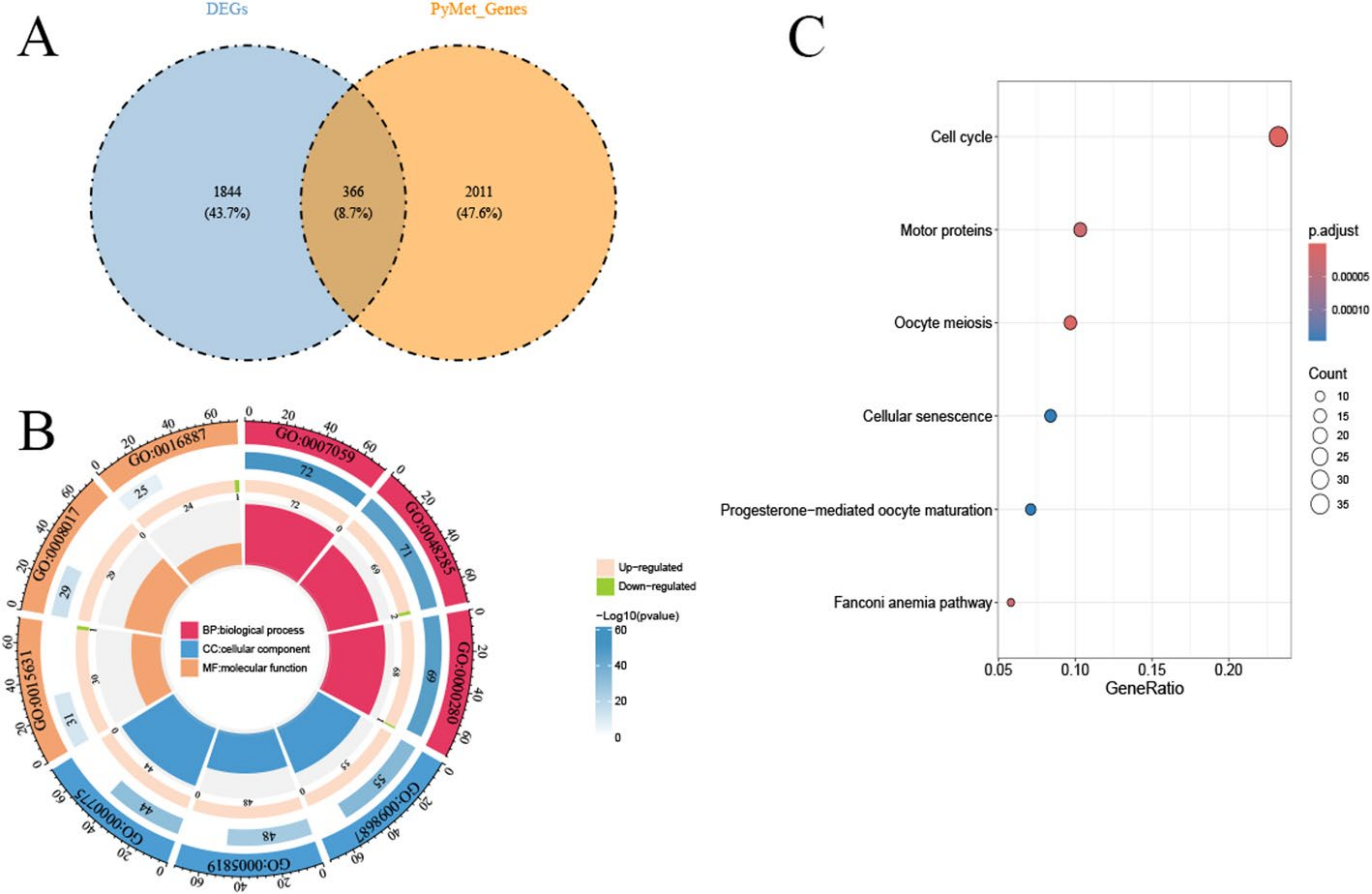
Enrichment results of candidate genes. **A** Screening of candidate genes. **B** GO enrichment analyses. **C** KEGG enrichment analyses

At the same time, a total of 11 genes passed the univariate cox (Fig. 1C) and PH assumption tests (Table 1). There was a remarkable disparity in survival between high (*n* = 87) and low (*n* = 34) scoring categories (optimal cut-off value of PyMRGs scores = 0.3863068), with the high-score category having a lower survival rate (*P* = 0.045) (Fig. 1D). The ssGSEA score of PyMRGs was taken as a trait to conduct WGCNA. No outlier samples were found in this step (Fig. 1E). The connectivity was close to 0, and soft threshold was chosen to be 4 (Fig. 1F). A total of 15 co-expression modules were identified based on the soft threshold (Fig. 1G). MEbrown (cor = 0.67, *P* = 6e-17) and MEyellow (cor = -0.67, *P* = 5e-17) were chosen as the key modules and 2377 genes in key modules were selected as PyMet-Genes based on the correlation coefficient (Fig. 1H). Finally, 2377 PyMet-Genes were intersected with 2210 DEGs to ascertain 366 candidate genes (Fig. 2A).

GO analysis of 366 candidate genes enriched a total of 560 results (Table S2), including 423 biological processes (BPs, e.g. chromosome segregation), 68 cellular components (CCs, e.g. condensed chromosome), and 69 molecular functions (MFs, e.g. tubulin binding). The top 3 pathways for each classification were demonstrated in graphs (Fig. 2B). Also, 23 KEGG signaling pathways (e.g. cell cycle) were enriched (Table S3), and the bubble diagram showed the top 6 signaling pathways (Fig. 2C).

### ECE2, NFE2L3, PFKFB3, FADS2, and SEPT3 were prognostic genes in TNBC

**Fig. 3:**
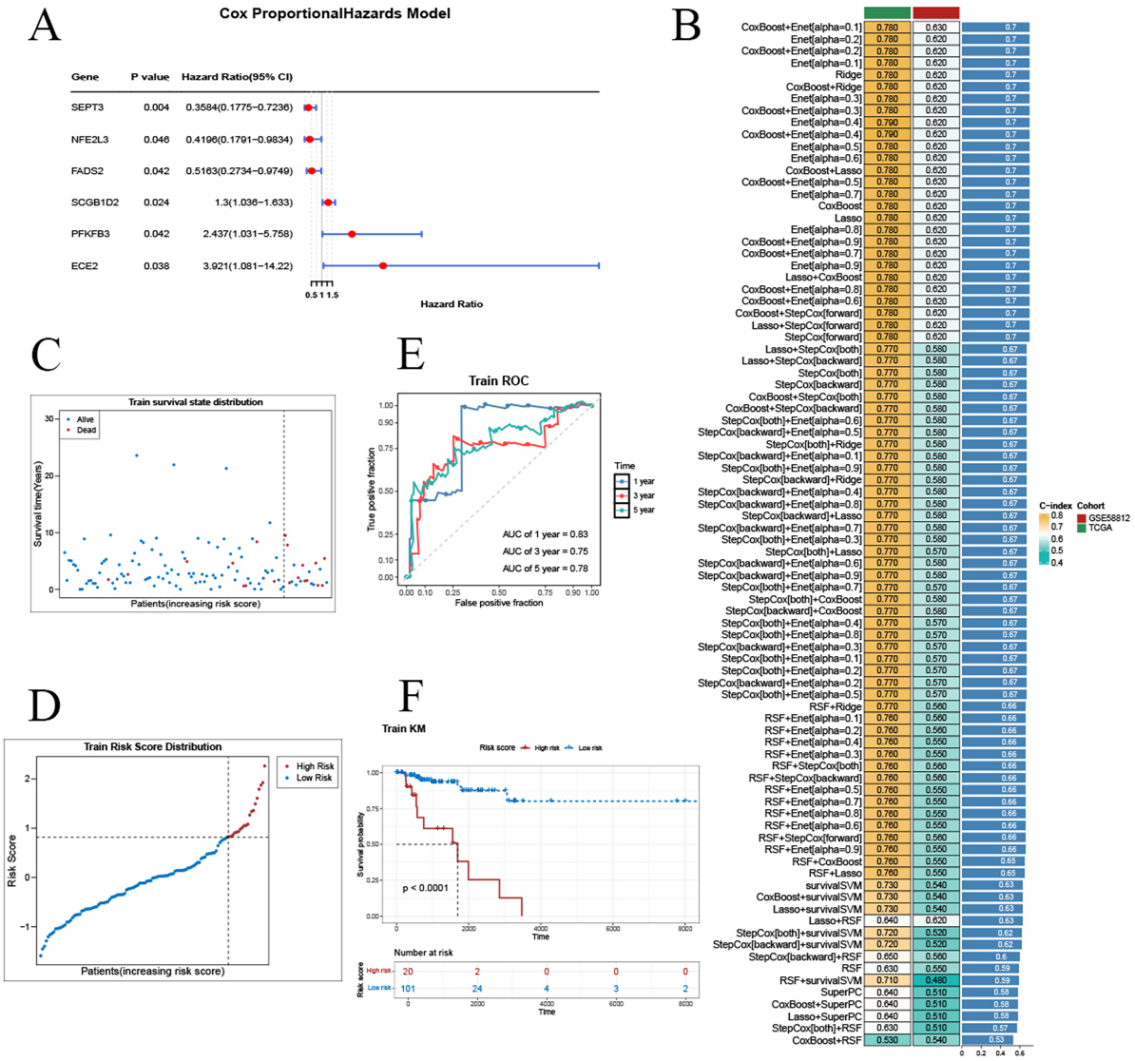
Prognostic gene screening and construction of the risk model. **A** The 5 candidate prognostic genes were identified via the implementation of univariate Cox analysis. **B** Screening 5 prognostic genes using 101 machine learning algorithms. **C** Graph of the distribution of risk scores in the TCGA - TNBC training set. **D** Graph of the distribution of survival status in the TCGA - TNBC training set. **E** K-M survival analysis of the TCGA - TNBC training set. **F** ROC curve analysis of the TCGA - TNBC training set

**Fig. 4:**
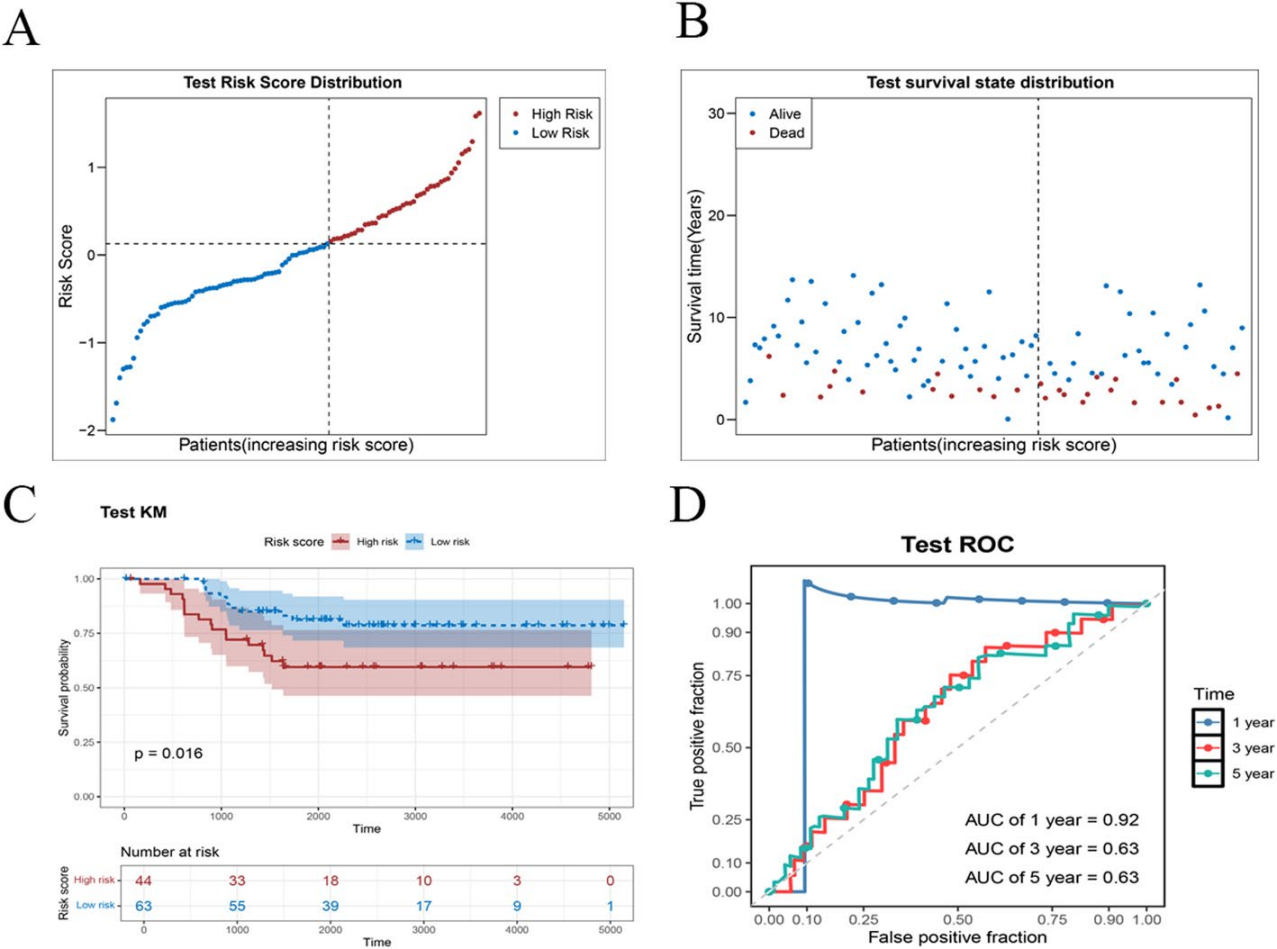
Validation of the risk model. **A** Graph of the distribution of risk scores of patients in the validation set. **B** Graph of the distribution of survival status of patients in the validation set. **C** K - M survival analysis of the validation set. **D** ROC curve analysis of the validation set

The 5 prognosis-related genes were identified via the implementation of univariate Cox analysis (HR ≠ 1 and *P* < 0.05) (Fig. 3A) and PH assumption test (*P* > 0.05) (Table 2). Subsequently, among 101 combinations of machine learning algorithms, CoxBoost+Enet[alpha = 0.1] model was determined to possess a concordance index (C-index) exceeding 0.6 in both the TCGA-TNBC (C-index = 0.780) and GSE58812 (C-index = 0.630) datasets (Fig. 3B). Concurrently, 5 prognostic genes (ECE2, NFE2L3, PFKFB3, FADS2, and SEPT3) were included in the model. The TNBC patients were partitioned into HRG and LRG groups in accordance with an optimal cutoff value of risk score, which was 0.8194215 for training set (HRG = 20, LRG = 101) (Fig. 3C) and 0.1284305 for validation set (HRG = 44, LRG = 63) (Fig. 4A). Furthermore, survival status graph demonstrated that a greater risk score corresponded to a larger number of dead TNBC patients (Figs. 3D and 4B). The K-M curves illustrated that increasing with years, survival probability of HRG patients was shorter than that of LRG patients (*P* < 0.05). In training set, P was less than 0.0001, while in validation set, p was equal to 0.016 (Figs. 3E and 4C). Moreover, in training set AUC values of ROC curves for 1-year, 3-year, and 5-year were 0.83, 0.75, and 0.78 respectively. In validation set, they were 0.92, 0.63, and 0.63, suggesting the risk model has good predictive performance for TNBC (Figs. 3F and 4D).

**Table 2:** Result of PH assumption test for prognostic genes

	chisq	df	*p*
ECE2	0.369945308655669	1	0.543034081113163
NFE2L3	0.084580363462138	1	0.771183608751042
PFKFB3	3.03187779157678	1	0.0816434779925847
FADS2	0.108055266633734	1	0.742369143094707
SEPT3	0.000114964491757288	1	0.991445126304337

### There was high accuracy in nomogram

**Fig. 5:**
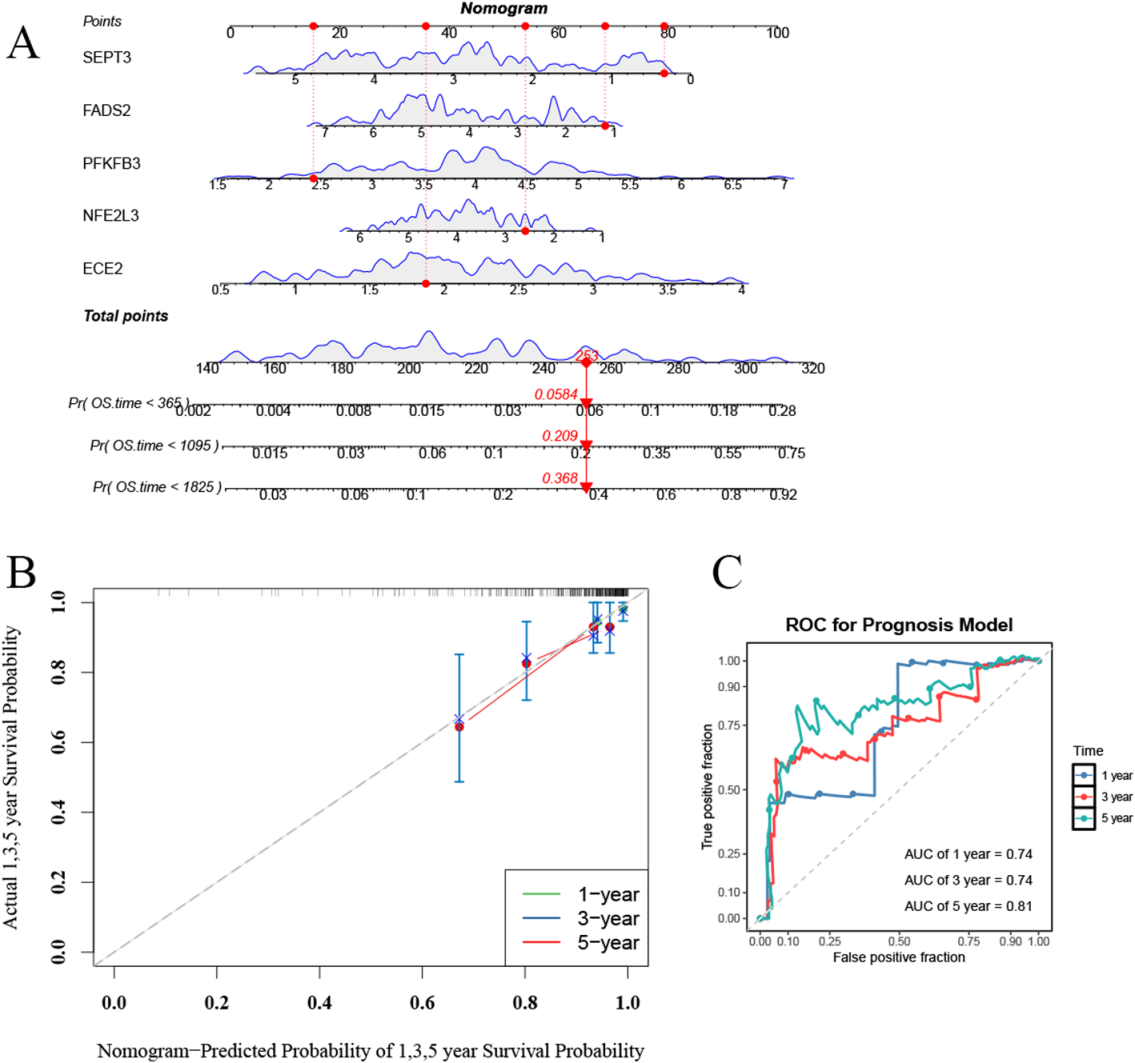
Nomogram construction. **A** Nomogram model constructed based on prognostic genes. **B** Calibration curve of the nomogram model. **C** The ROC curve of the nomogram

The higher total score of prognostic genes on the nomogram was associated with lower 1, 3, 5-year survival rates of TNBC patients (Fig. 5A). The calibration curve exhibited a slope of approximately 1, which signified that the nomogram was characterized by a high degree of accuracy in prognostic prediction (Fig. 5B). AUC values for the 1, 3, 5-year ROC curves of nomogram were all above 0.7 (Fig. 5C). It indicated that the nomogram had a relatively strong ability to predict prognosis.

### Multiple TFs and pathways were related to the prognosis of TNBC patients

**Fig. 6:**
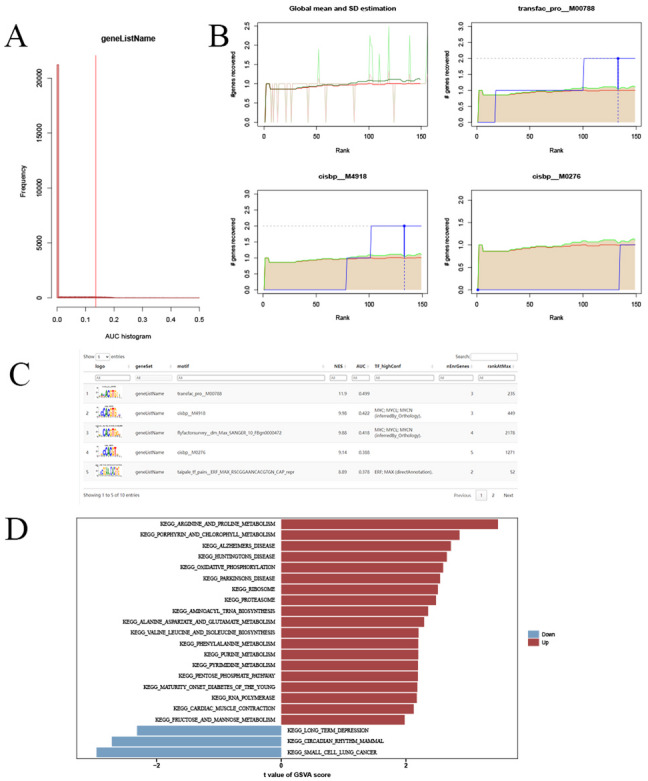
GSVA analysis and enrichment analysis of TF motifs. **A** Histogram of the AUC. **B** Cumulative recovery curve of significantly enriched motifs. **C** Motifs enriched with prognostic genes and the corresponding major transcription factors. **D** GSVA analysis

TF motif prediction was used to explore the co-regulatory mechanisms of prognostic genes. Cumulative recovery curves were employed to enrich these transcription factors (Fig. 6A, B). The analysis results showed that the transfac_pro_M00788 (NES = 11.9, AUC = 0.499) and cisbp_M4918 (NES = 9.98, AUC = 0.422) motifs were significantly enriched in the regulatory regions of prognostic genes. Multiple TFs of the prognostic genes were identified (Fig. 6C).

GSVA ascertained a total of 22 significantly different pathways between HRG and LRG, of which 19 pathways (e.g. arginine and proline metabolism) were activated and 3 pathways (such as circadian rhythm mammal) were inhibited in the HRG (Fig. 6D).

### Missense mutation was the main type of mutation in TNBC patients

**Fig. 7:**
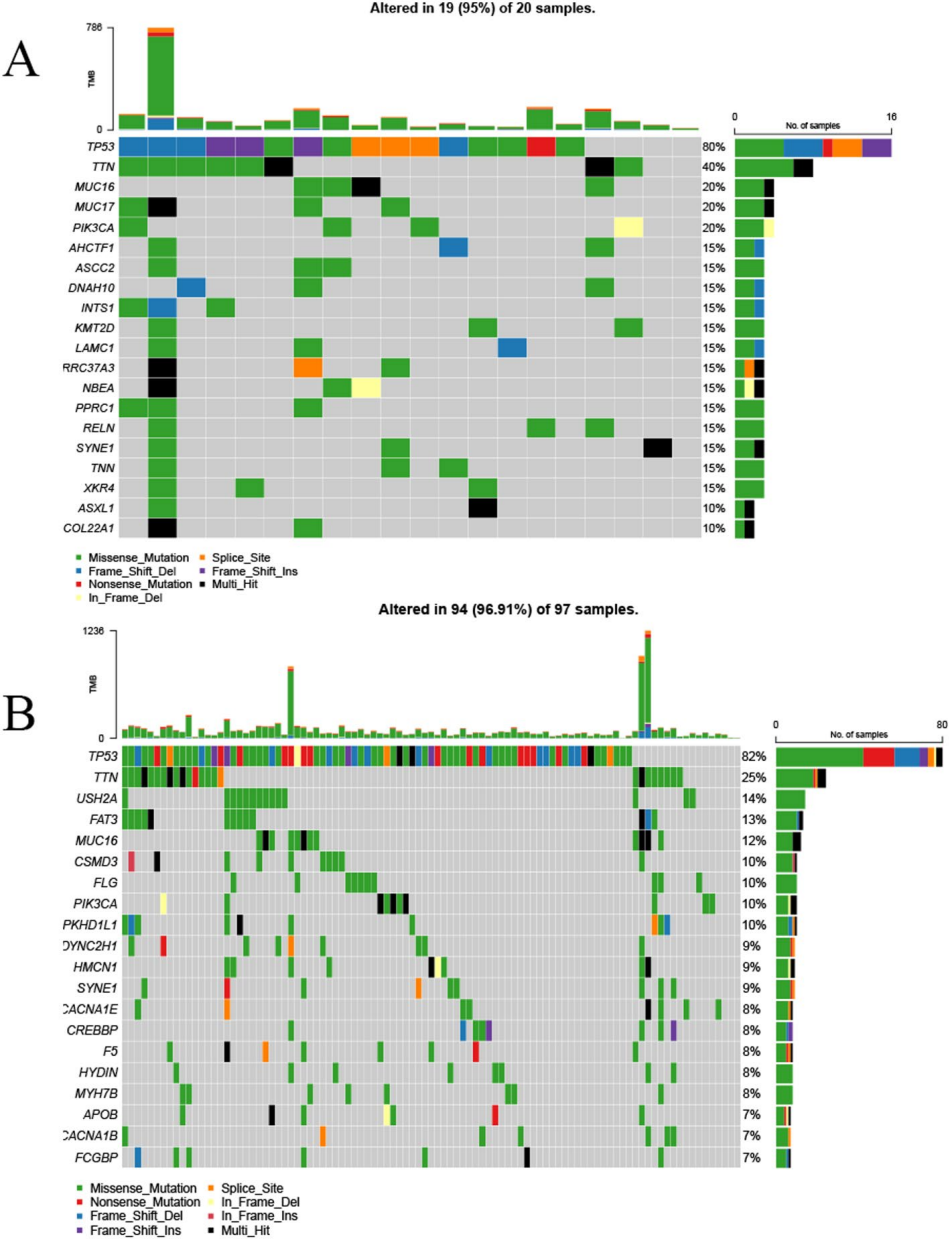
Analysis of the mutation landscape. **A** Mutation landscape of the HRG group in the training set. **B** Mutation landscape of the LRG group in the training set

The somatic mutation analysis of TNBC patients revealed that missense mutations were the predominant mutation type (Fig. 7A, B). Compared with HRG, the LRG exhibited a higher proportion of mutations. Among them, the 3 genes with the highest mutation frequencies in LRG were TP53 (82%), TTN (25%), and USH2A (14%). The number of samples with TP53 gene mutations was 16. Among them, 5 samples were found to be with missense mutations, 4 samples were identified with frameshift deletion mutation (Fig. 7A). In contrast, in LRG, TP53 (80%), TTN (40%), and MUC16 (20%) was top 3 genes with highest mutation frequencies. Among them, the number of samples with TP53 gene mutations was 80. Among these samples, 42 were found to have missense mutations, and 15 were found to have nonsense mutations (Fig. 7B). The increased mutation frequency of MUC16 in HRG might have indicated its association with the prognosis of TNBC patients.

### Epithelial cells were related to the prognostic genes

**Fig. 8:**
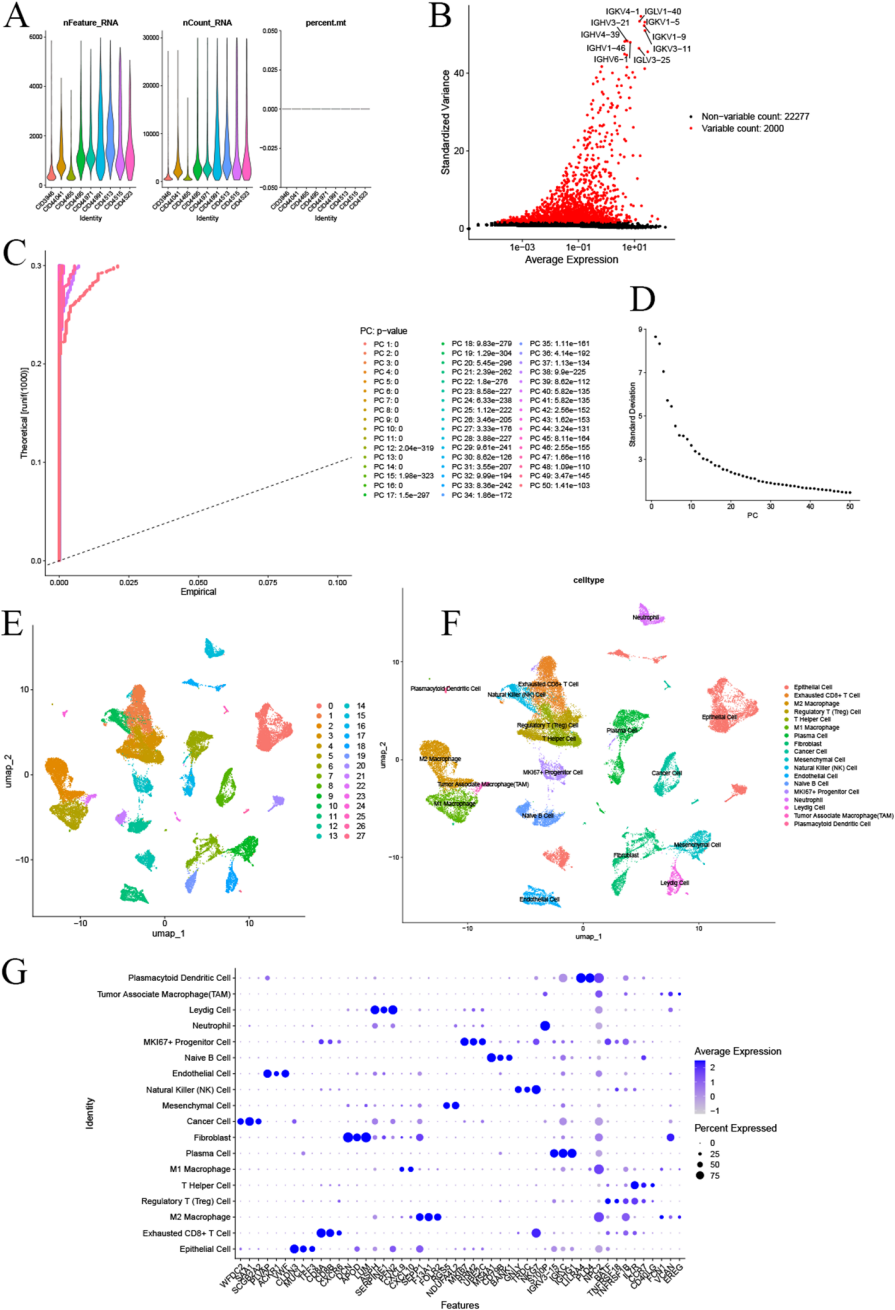
Single-cell data analysis. **A** Screening of highly variable genes. **B** PCA of highly variable genes. **C** Identification of available dimensions. **D** Dimensionality reduction and clustering using UMAP. **E** UMAP graph of cell clustering. **F** Bubble plot of the expression of marker genes in different cell populations. **G** Dot plot showing the expression levels of marker genes across annotated cell types

After data filtering, 37,278 cells and 24,277 genes were obtained from 9 samples, and the top 2000 highly variable genes (such as HP, and KCNJ2) were selected for PCA (Fig. 8A, B). Based on the PCA replacement test and the scree plot, the top 30 PCs were selected for cell cluster analysis (Fig. 8C, D). In the end, 28 cell clusters were obtained and annotated as 18 cell types, such as cancer cells and endothelial cells (Fig. 8E, F). Moreover, the marker genes of the cell clusters were all characterized by specificity (Fig. 8G).

**Fig. 9:**
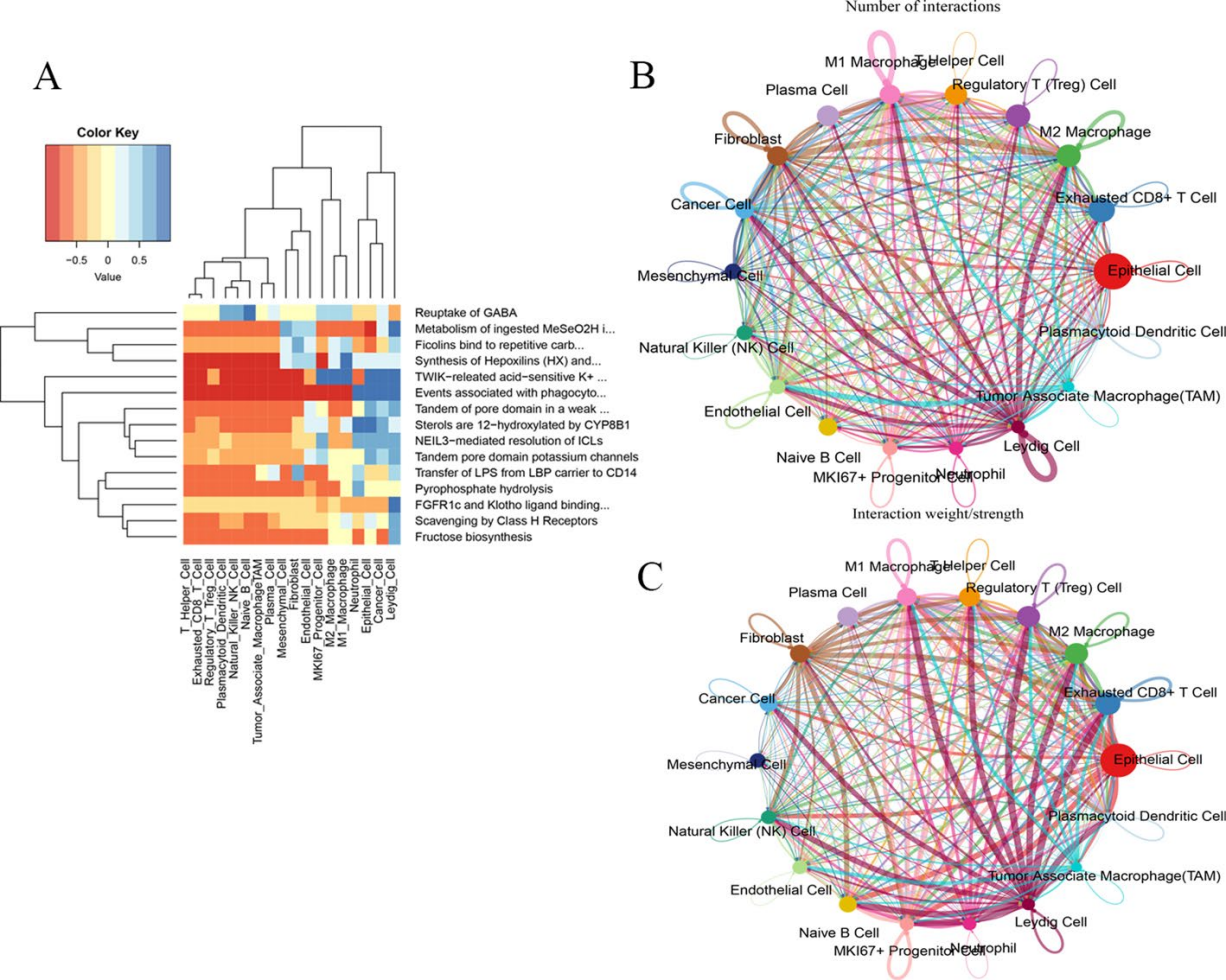
Enrichment analysis of cells and cell communication. **A** Functional enrichment analysis of cells. **B** Cell communication diagram of the annotated cells in the disease group, where the thickness of the connecting lines represents the number of interactions between cells. **C** Cell communication diagram of the annotated cells in the disease group, where the thickness of the connecting lines represents the intensity of the interaction between cells

**Fig. 10:**
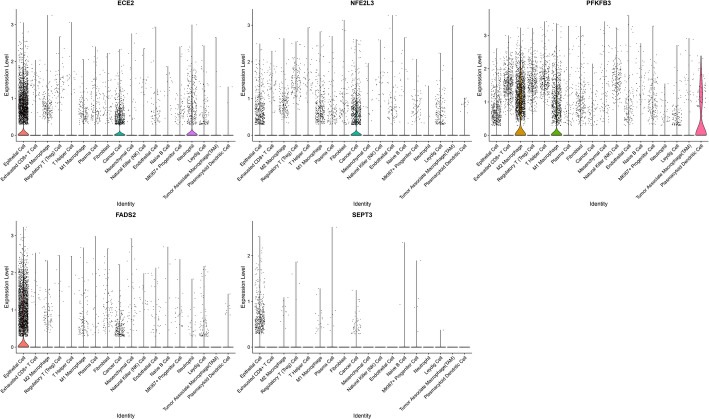
The expression levels of prognostic genes in different cells

A total of 1733 pathways (such as reuptake of gamma-Aminobutyric acid (GABA)) were enriched for 18 cell types (Table S4), and the top 15 pathways were illustrated in Fig. 9A. The results of cell communication indicated that leydig cells had the largest number of interactions with other cells and the strongest intensity (Fig. 9B, C). Furthermore, ECE2, NFE2L3, PFKFB3, FADS2, and SEPT3 were all expressed in epithelial cells, which indicated that epithelial cells were related to the prognosis in TNBC (Fig. 10). In conclusion, epithelial cells might play an important role in TNBC through the metabolism of ingested MeSeO_2_H into MeSeH and TWIK-related acid-sensitive K⁺ channel (TASK) pathways (Table 3).

### The sensitivity differences of 12 drugs between the two risk groups and the molecular docking of prognostic genes with drugs.

**Fig. 11:**
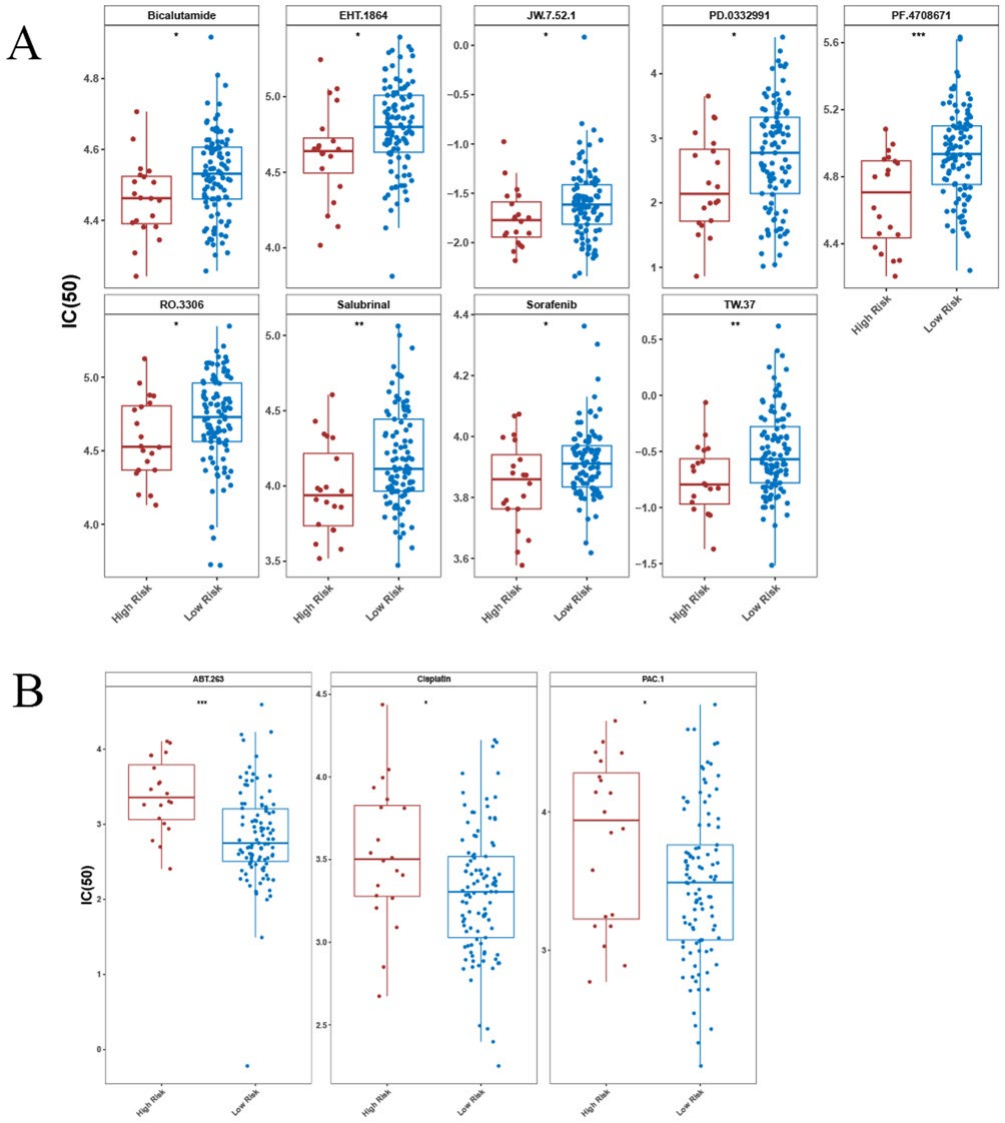
The sensitivity of the high-risk and low-risk groups to chemotherapeutic drugs. **A** The difference in IC50 of drugs with downregulated expression between the high-risk and low-risk groups. **B** The difference in IC50 of drugs with upregulated expression between the high-risk and low-risk groups

There were 138 drugs predicted in the database, of which 12 exhibited remarkable disparity in IC_50_ between HRG and LRG (Table S5). The figure displayed 9 drugs that were sensitive to HRG (Fig. 11A) and 3 drugs that were sensitive to the LRG (Fig. 11B). For example, the HRG exhibited sensitivity to PD-0332991, while the LRG demonstrated sensitivity to ABT-263. In addition, the results of the molecular docking showed that there was a relatively strong binding ability between the prognostic genes and the drugs, and all the binding energies were less than − 5 kJ/mol (Table 3). For example, the binding energy between PFKFB3 and ABT.263 was − 8.2 kJ/mol, and the binding energy between FADS2 and R0.3306 was − 7.2 kJ/mol (Figure S2A-E).

**Table 3:** The result of molecular docking

Gene name	Drug	Vina_score (kJ mol)
ECE2	Cisplatin	-5.7
NFE2L3	PF.4,708,671	-6.3
PFKFB3	ABT.263	-8.2
FADS2	R0.3306	-7.2
SETP3	TW.37	-6.6

### The expression of prognostic genes was validated in clinical samples

**Fig. 12:**
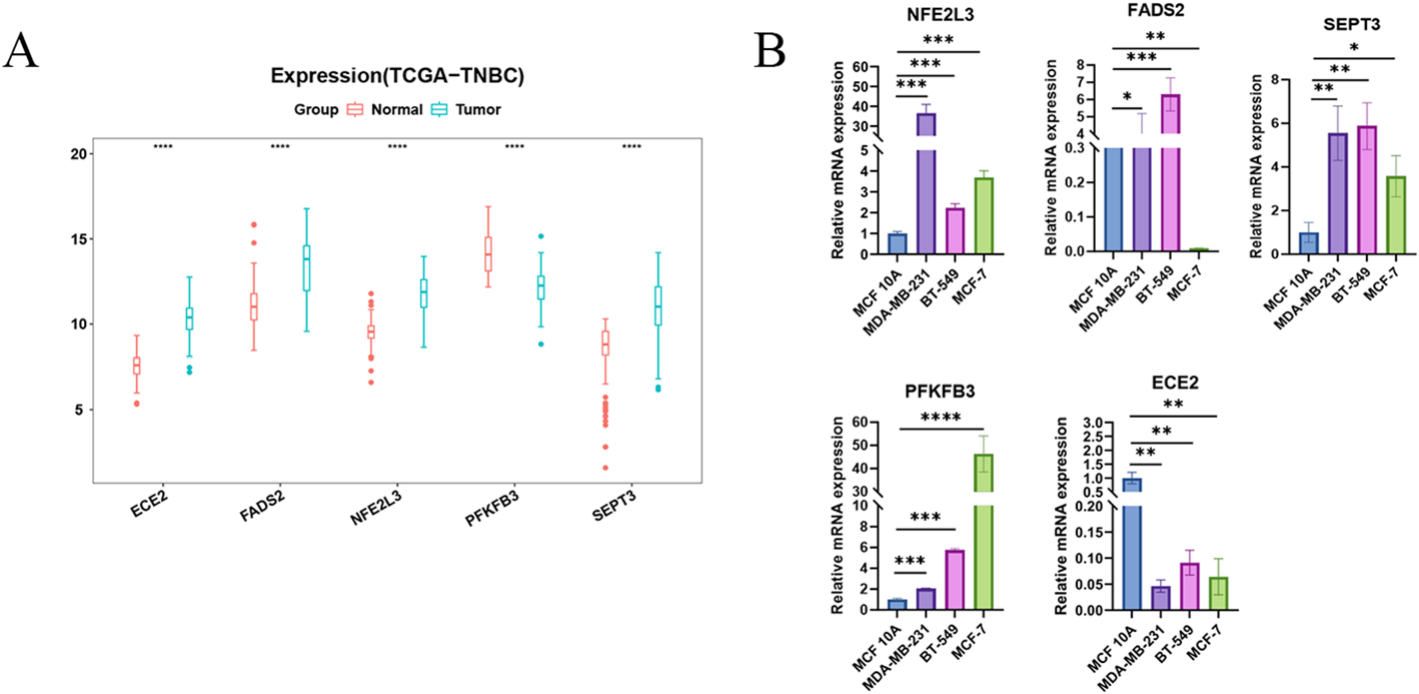
The expression status of prognostic genes. **A** The expression of prognostic genes in TCGA-TNBC. **B** Differential expressions of prognostic genes in normal human breast cells and BC cell lines

In TCGA-TNBC, compared with control group, the expression of ECE2, NFE2L3, FADS2 and SEPT3 were remarkably elevated, while expression of PFKFB3 was remarkably decreased in TNBC group (*P* < 0.05) (Fig. 12A). qPCR results showed that compared with normal human breast cells, NFE2L3, FADS2, PFKFB3, and SEPT3 were significantly upregulated in BT-549 and MDA-MB-231 cell lines (*P* < 0.05), while ECE2 showed no significant change. Notably, PFKFB3 exhibited a more pronounced upregulation in the Luminal A breast cancer cell line MCF7 (Fig. 12B, Table S7). All primers exhibited a single, sharp melting peak, with no additional peaks or plateaus, indicating high primer specificity and reliable qPCR detection results (Figure S3).

### Validation of the functional role of SEPT3 in regulating proliferation, migration, and invasion of TNBC cells

**Fig. 13:**
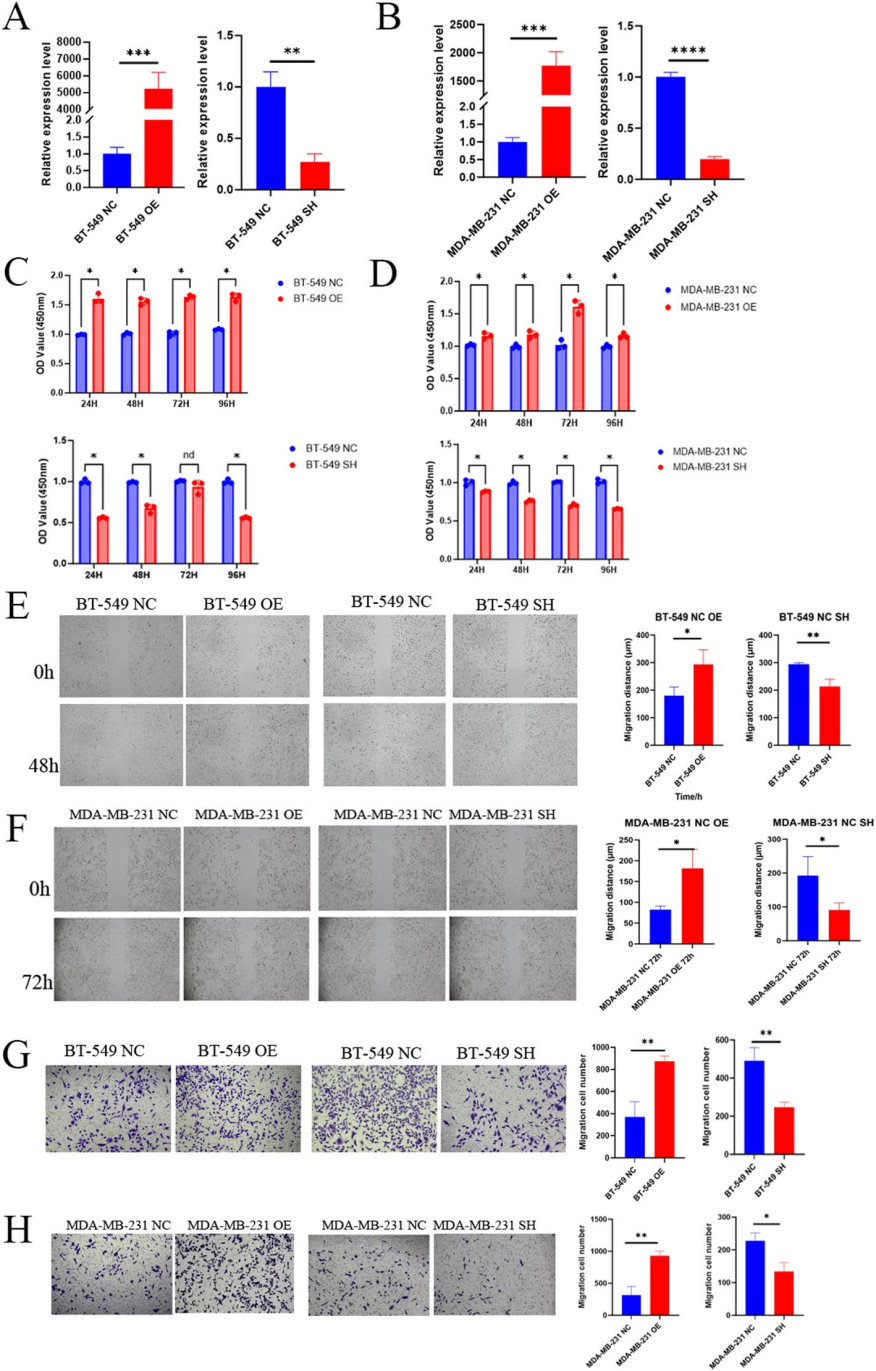
SEPT3(a key PyMRG): Regulation of TNBC Cell Proliferation, Migration, and Invasion **A** Relative expression of SEPT3 in BT-549 cells with OE, SH, or NC. **B** Relative expression of SEPT3 in MDA-MB-231 cells with OE, SH, or NC. **C** Proliferation of BT-549 cells (OE, SH, or NC) assessed by CCK-8 assay at 24–96 h. **D** Proliferation of MDA-MB-231 cells (OE, SH, or NC) assessed by CCK-8 assay at 24–96 h. **E** Wound healing assay for migration of BT-549 cells (OE, SH, or NC) at 0 h and 48 h; quantification of migration distance. **F** Wound healing assay for migration of MDA-MB-231 cells (OE, SH, or NC) at 0 h and 72 h; quantification of migration distance. **G** Transwell invasion assay of BT-549 cells (OE, SH, or NC); quantification of invaded cell number. **H** Transwell invasion assay of MDA-MB-231 cells (OE, SH, or NC); quantification of invaded cell number

This study systematically investigated the functional role of SEPT3 in breast cancer cell biology using two TNBC cell lines (BT-549 and MDA-MB-231) with three genetic manipulation strategies (SEPT3 OE, SH, and NC). First, genetic modification efficiency was validated: quantitative reverse transcription-polymerase chain reaction (qRT-PCR) (Fig. 13A, B) confirmed that SEPT3 expression was significantly upregulated in OE cells and downregulated in SH cells relative to NC controls, ensuring reliable experimental conditions. Subsequent functional assays consistently demonstrated the regulatory effects of SEPT3 on key malignant phenotypes of TNBC cells across both cell lines: (1) Cell proliferation (CCK-8 assays, Fig. 13C, D: SEPT3 overexpression enhanced time-dependent cell proliferation, while SEPT3 knockdown suppressed proliferative capacity; (2) Cell migration (wound healing assays, Fig. 13E, F): OE cells exhibited accelerated wound closure (reflected by longer migration distance), whereas SH cells showed impaired migratory ability compared with NC controls; (3) Cell invasion (Transwell invasion assays, Fig. 13G, H): SEPT3 overexpression increased the number of invaded cells, while its knockdown reduced invasive potential. Collectively, these in vitro data indicate that SEPT3 promotes the proliferation, migration, and invasion of TNBC cells.

## Discussion

Over the past few decades, the treatment and survival rates for BC have improved significantly [49, 50]. However, due to a lack of specific therapeutic targets, there is currently no targeted standard treatment regimen for TNBC, and it is also difficult to predict the prognosis of TNBC patients. PyM involves nucleoside salvage, de novo nucleotide synthesis, and the catalytic degradation of pyrimidines [51]. Research has shown that a stable supply of deoxynucleoside triphosphates (dNTPs) is critical for cancer cells [52, 53]. Activation of PyMRGs is therefore considered a necessary condition for tumor growth [54]. Nevertheless, the prognostic significance of PyMRGs in TNBC remains to be elucidated. In this study, TNBC data from the Xena and GEO databases were used, and combined with 101 machine learning algorithms to screen out five prognostic genes (ECE2, NFE2L3, PFKFB3, FADS2, and SEPT3) and construct a highly accurate prognostic model. This model was validated in the single-cell dataset GSE176078.

ECE2 (Endothelin-converting enzyme 2) is an enzyme involved in the metabolism of endothelin [55]. Its expression is significantly higher in basal-like breast cancer than in other PAM50 subtypes. The mRNA and protein expressions in clinical BC tissues are higher than in adjacent non-cancerous tissues. ECE2 promotes breast cancer progression by upregulating E2F1 and MYC, and its high expression is associated with poor prognosis [56].

PFKFB3 (6-phosphofructo-2-kinase/fructose-2,6-bisphosphatase 3) is a key enzyme in glycolysis. In the context of BC, PFKFB3 plays a crucial and multifaceted role in the processes of tumor initiation, progression, and the development of drug resistance [57]. In TNBC, we have found that the PFKFB3 gene exhibits a characteristic of significant downregulation of expression. The downregulation of PFKFB3 may affect the prognosis of TNBC by remodeling the tumor metabolic network. As a core metabolic feature of tumor cells, glycolysis may be inhibited by the reduced expression of PFKFB3, which promotes tumor cells to switch metabolic pathways and change the metabolic product profile, thereby affecting the tumor microenvironment, tumor cell proliferation, and immune escape [58]. In addition, the downregulation of PFKFB3 may also intervene in the processes of tumor cell proliferation, apoptosis, and autophagy by regulating downstream signaling pathways, such as the PI3K/AKT pathway. Thus, it plays a crucial role in the progression and prognosis of TNBC [59].

FADS2 (Fatty acid desaturase 2) plays a crucial role in fatty acid metabolism. It converts ω-6 precursors into long-chain unsaturated fatty acids associated with cell membranes and the nervous system [60]. FADS2 regulates lipid metabolism and ferroptosis sensitivity in TNBC [61]. Inhibiting FADS2 and its downstream pathways can reduce the proliferation and migration ability of cancer cells [62].

Septin 3 (SEPT3), which is an integral member of the septin family, plays a significant role in the assembly of the cytoskeleton and is responsible for maintaining the shape and polarity of cells [63]. Specifically, SEPT3 exerts its influence by promoting the invasion and proliferation of TNBC cells through the activation of the Wnt signaling pathway. Furthermore, SEPT3 has been identified as an independent risk factor for TNBC, and its presence is closely associated with the TNM staging system, as well as the occurrence of lymph node metastasis in patients with this particular subtype of breast cancer, highlighting its importance in the pathophysiology and clinical prognosis of TNBC [64].

NFE2L3 (Nuclear factor erythroid 2 (NF-E2)-related factor 3) is a nuclear transcription factor [65]. It is highly expressed in BC, particularly TNBC. NFE2L3 inhibits the metastasis and proliferation of TNBC cells by suppressing ERK activation in a ROS-dependent manner. Its deficiency leads to over-activation of the ERK pathway, promoting tumor invasion and metastasis [66, 67].

Previous research has demonstrated that the five prognostic genes screened via 101 machine-learning algorithms are all closely associated with the development and progression of BC. This further validates the reliability of the prognostic model, suggesting that the model offers a novel possibility for predicting the prognosis of TNBC patients.

GSVA analysis revealed that 19 pathways were activated in the high-risk group, while 3 were inhibited. Among these, studies have shown that arginine and proline metabolism play a crucial role in breast cancer. PYCR1, a key enzyme in proline synthesis, is highly expressed in TNBC and is closely associated with malignant progression [68]. PYCR1 promotes the proliferation and invasion of tumor cells by catalyzing the conversion of δ1-pyrroline-5-carboxylic acid (P5C) into proline [69] .

Meanwhile, single-cell analysis reveals that the expression of prognostic genes is related to epithelial cells. A number of studies have shown the relationship. For example, the TGF-β-induced epithelial-to-mesenchymal transition (EMT) pathway [70]. The TGF-β signaling pathway can prevent the degradation of Snail protein by chaperone-mediated autophagy (CMA), thereby promoting the occurrence of EMT and playing a significant role in TNBC [71]. Additionally, TGF-β regulates the proliferation, migration, and invasion of TNBC cells through other mechanisms [72].

Based on the expression levels of prognostic genes from single-cell analysis, as well as the results of cell communication and cell enrichment, prognostic genes are predominantly distributed in epithelial cells. The epidermal growth factor receptor (EGFR) is extensively present on the surface of epithelial cells. The EGFR signaling pathway exerts a crucial influence on physiological processes including cell growth, proliferation, and differentiation. It is highly expressed in most TNBC cases [73, 74].

Based on the comprehensive findings of the present study, SEPT3 emerges as the optimal candidate for the key pyrimidine metabolism-related gene in TNBC, attributed to its distinct advantages across multi-dimensional analyses. Firstly, among the five candidate genes, SEPT3 is the sole one that concurrently fulfills the dual criteria of “high statistical significance + stable predictability” as a TNBC prognostic indicator. Univariate Cox regression analysis confirms it as an independent prognostic protective factor with no time-dependent bias. Secondly, SEPT3 acts as the core supportive gene in prognostic machine learning models: models integrated with SEPT3 demonstrate significantly superior predictive performance compared to those associated with other genes, and the predictive efficacy of the models decreases substantially following the exclusion of SEPT3. Thirdly, SEPT3 presents a unique expression profile featured by “significant differential expression between tumor and normal tissues + strong correlation with risk stratification,” displaying stable and remarkable expression differences in both clinical samples and cell lines, while other genes only show differential expression in specific contexts. Fourthly, regarding functional relevance, SEPT3 is predominantly localized in TNBC epithelial cells and shows a significant correlation with EMT phenotypic markers [75]. Given the intimate link between EMT and tumor cell metabolic reprogramming (including pyrimidine metabolism), this further underscores the potential core role of SEPT3 in regulating tumor metabolism. Supported by the aforementioned all-round evidence, SEPT3 is therefore selected as the key pyrimidine metabolism-related gene for subsequent experimental validation.

This study investigated the functional role of SEPT3 in TNBC using two cell lines (BT-549, MDA-MB-231) with three genetic manipulations: SEPT3 OE, SH, and NC. In conclusion, the current in vitro functional data confirm SEPT3 as a driver of TNBC cell proliferation, migration, and invasion, and lay the foundation for exploring its mechanistic links to EMT and pyrimidine metabolism. Future in vivo and molecular studies will further validate its clinical value as a prognostic biomarker and therapeutic target in TNBC.

Between the high-risk group and the low-risk group, we identified 12 drugs with significant differences in IC50 values, highlighting the existence of different drug responses based on patient risk stratification. A lower IC50 value indicates that a lower concentration of the drug is required to inhibit biological activity, meaning that cells or tissues are more sensitive to the drug. Conversely, a higher IC50 value indicates that cells or tissues have poor sensitivity to the drug [76]. PD-0332991 showed sensitivity in the high-risk group, while ABT-263 was more effective in the low-risk group. This suggests that customizing drug selection according to the patient’s risk group can optimize treatment outcomes. Since triple-negative breast cancer lacks estrogen, progesterone, and human epidermal growth factor receptor 2 (HER2) and there is no specific targeted treatment, this personalized drug strategy is particularly crucial [77]. Through molecular docking, it was found that the binding energies between prognostic genes (such as PFKFB3, FADS2, etc.) and their corresponding drugs were all less than − 5 KJ/Mol, indicating a strong binding affinity. For example, the binding energy between PFKFB3 and ABT-263 reached − 8.2 KJ/Mol, and the binding energy between FADS2 and R0.3306 was − 7.2 KJ/Mol, suggesting that these gene-drug pairs have great potential in the treatment of TNBC and may exert therapeutic effects through specific binding. Taking the PFKFB3 gene as an example, its expression is downregulated in TNBC, and this characteristic may enable it to play a key role in the mechanism of action of ABT-263. Changes in gene expression can alter the intracellular environment and metabolic pathways, affecting the targets and processes of drug action [78]. For instance, the downregulation of PFKFB3 may change the metabolic network of tumor cells, resulting in different modes and effects of ABT-263, suggesting that drugs more effective for patients can be screened based on the gene expression status [79]. In-depth understanding of the mechanism of gene-drug interactions provides an important direction and basis for revealing the pathogenesis of tumors, optimizing treatment strategies, and improving patient prognosis.

This study obtained five prognostic genes based on the transcriptomic data of TNBC. These five genes can provide new ideas for improving the treatment of TNBC. However, there are still some limitations in our research. For example, the dataset in this study has a limited sample size, and there is an imbalance in the proportion of the number of survivors and the number of deaths in the sample, with a relatively low mortality rate. This uneven data distribution may lead to overly wide confidence intervals for the hazard ratios (HR) of some genes. In the future, the sample size should be expanded, more patients should be included, the proportion of surviving and deceased samples should be balanced, the representativeness of the data should be enhanced, and the impact of sampling errors on the results should be reduced. We will conduct additional functional experiments (e.g., validation related to pyrimidine metabolism) to further investigate the specific mechanisms underlying the roles of these genes in TNBC. We shall persistently monitor the functions of these genes, related pathways, and drugs.

## Conclusion

This study utilized TNBC datasets from the Xena and GEO databases, along with pyrimidine metabolism-related genes from online databases. Through WGCNA, differential expression analysis, univariate Cox regression, and 101 combinations of machine learning algorithms, five prognostic genes (ECE2, NFE2L3, PFKFB3, FADS2, and SEPT3) were identified. A highly accurate prognostic model was then constructed. The model was validated in the GSE58812 dataset. By leveraging the optimal cutoff value for risk scores, the disease samples were segregated into two distinct groups, namely the high-risk group and the low-risk group. Subsequently, a nomogram was meticulously constructed on the basis of the identified prognostic genes, and the calibration curves as well as the ROC curves were utilized to demonstrate that the nomogram possessed a high degree of predictive accuracy. Furthermore, by validating the functional role of SEPT3—a key pyrimidine metabolism-related gene—in regulating the proliferation, migration, and invasion of cells, we aim to confirm the validity of the model in the context of TNBC. This comprehensive approach and the resulting findings offer a novel and promising opportunity for the prediction of the prognosis of patients suffering from TNBC, thereby potentially facilitating more informed clinical decision-making and personalized treatment strategies.

## Supplementary Information

Below is the link to the electronic supplementary material.

Supplementary Material 1.

Supplementary Material 2.

Supplementary Material 3.

Supplementary Material 4.

## Data Availability

The datasets generated and/or analysed during the current study are available in public repositories. The TCGA-TNBC gene expression profiles and survival data are available from the UCSC Xena database (https://xena.ucsc.edu/). The GSE58812 and GSE176078 datasets are available from the Gene Expression Omnibus (GEO) repository: https://www.ncbi.nlm.nih.gov/geo/query/acc.cgi?acc=GSE58812 and https://www.ncbi.nlm.nih.gov/geo/query/acc.cgi?acc=GSE176078. All data used in this study are publicly available.

## Abbreviations

TNBC: Triple-negative breast cancer
BC: Breast cancer
WGCNA: Weighted gene co-expression network analysis
GSVA: Gene set variation analysis
HER2: Human epidermal growth factor receptor 2
PyMRGs: Pyrimidine metabolism related genes
UBE2T: Ubiquitin-conjugating enzyme E2T
CAD: Carbamoyl-phosphate synthetase 2/aspartate transcarbamylase/dihydroorotase
scRNA-seq: Single-cell RNA sequencing
PyM: Pyrimidine metabolism
TCGA: The cancer genome atlas
GEO: Gene expression omnibus
FC: Fold change
DEGs: Differentially expressed genes
KEGG: Kyoto encyclopedia of genes and genome
GO: Gene ontology
LOOCV: Leave-one-out cross-validation
LASSO: Least absolute shrinkage and selection operator
SVM: Support vector machine
K-M: Kaplan-meier
ROC: Receiver operating characteristic
NES: Normalized enrichment score
AUC: Area under the curve
TFs: Transcriptional factors
MSigDB: Molecular signatures database
ssGSEA: Single-sample gene set enrichment analysis
PCA: Principal component analysis
UMAP: Uniform manifold approximation and projection
GDSC: Genomics of drug sensitivity in cancer
IC50: Half-maximal inhibitory concentrations
dNTPs: Deoxynucleoside triphosphates
ECE2: Endothelin-converting enzyme 2
PFKFB3: 6-phosphofructo-2-kinase/fructose-2,6-bisphosphatase 3
FADS2: Fatty acid desaturase 2
SEPT3: Septin 3
NFE2L3: Nuclear factor erythroid 2 (NF-E2)-related factor 3
EMT: Pithelial-to-mesenchymal transition
CMA: Chaperone-mediated autophagy
EGFR: Epidermal growth factor receptor
ICD: Immunogenic cell death
