# Supplementary figures and images for: Integrating single-cell RNA and bulk RNA sequencing data to identify prognostic genes associated with pyrimidine metabolism in triple-negative breast cancer by machine learning algorithm combinations
Source: Discov Oncol. 2026 Apr 30;17:916. doi: 10.1007/s12672-026-05118-6 (PMC13275962; doi:10.1007/s12672-026-05118-6)

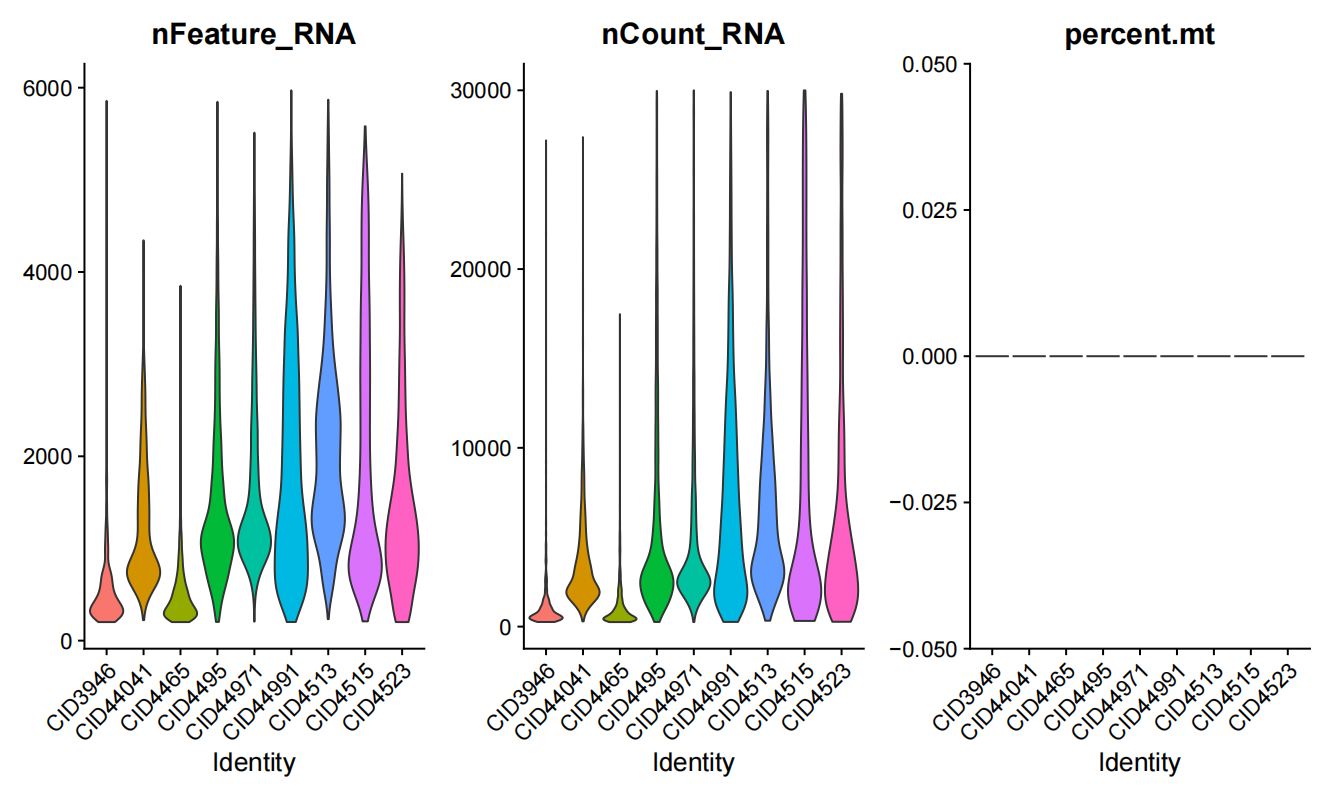

Supplement: Supplementary file 1 — Supplementary Material 1. [file 12672_2026_5118_MOESM1_ESM.png]

A1
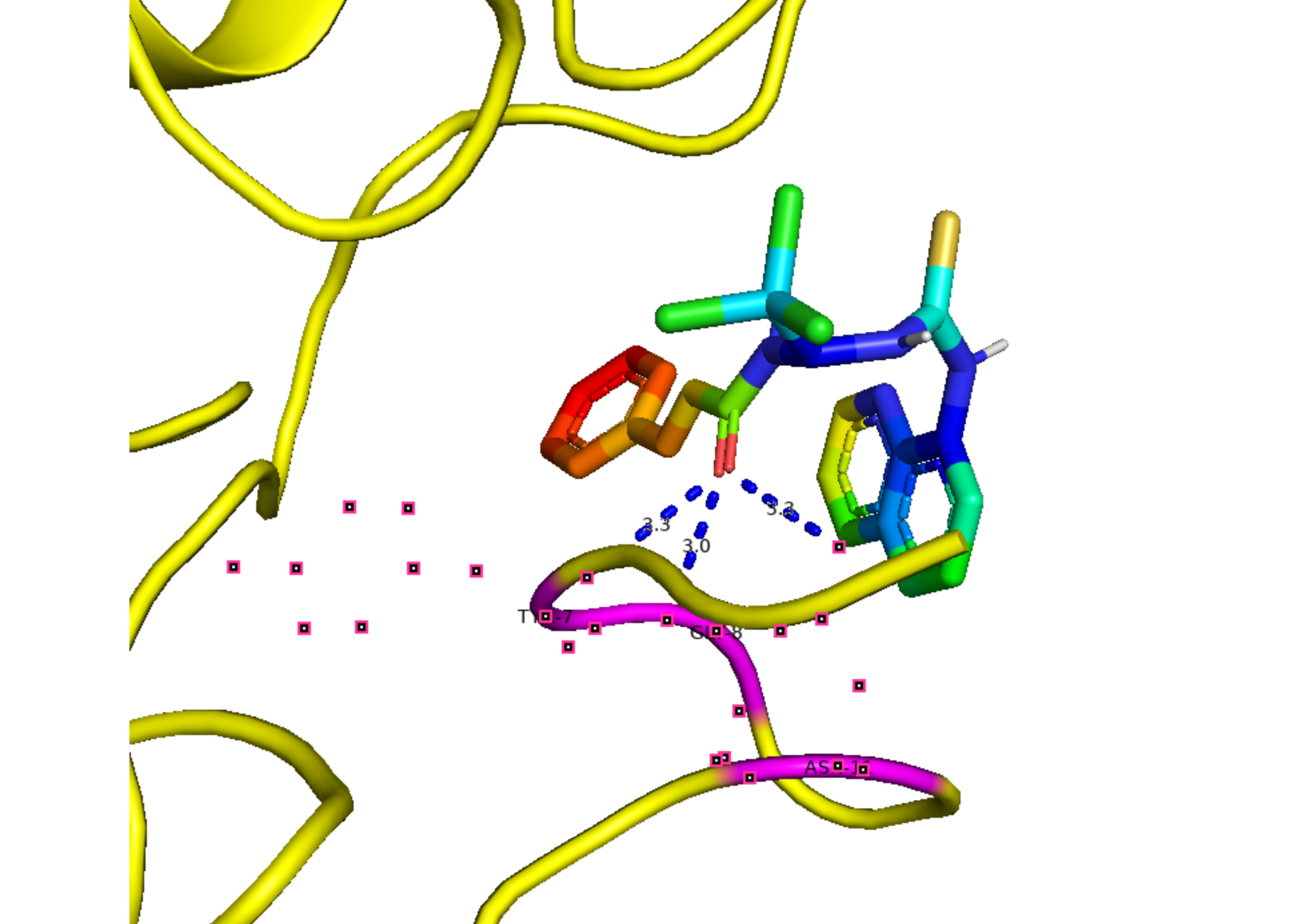


A2


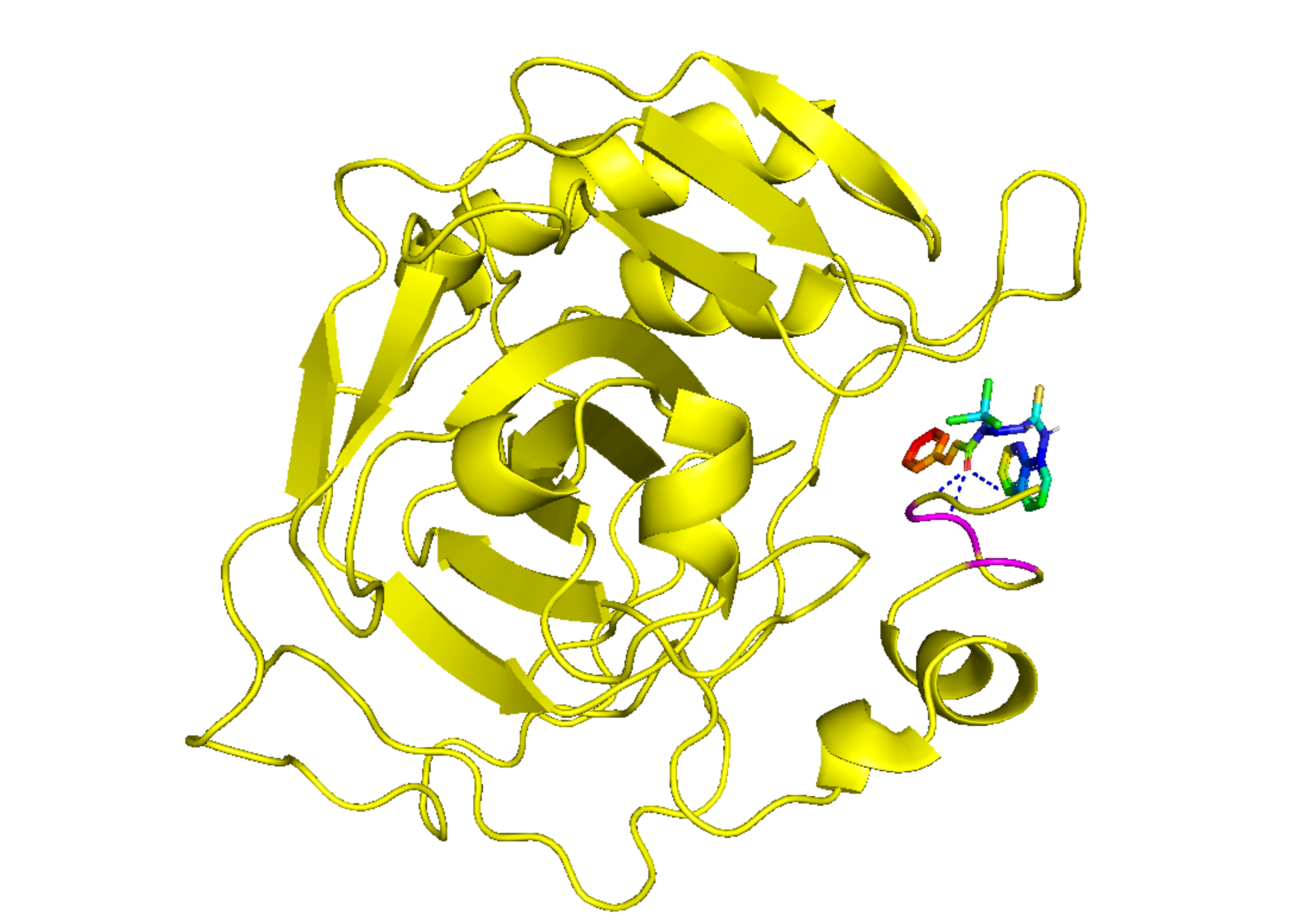


B1


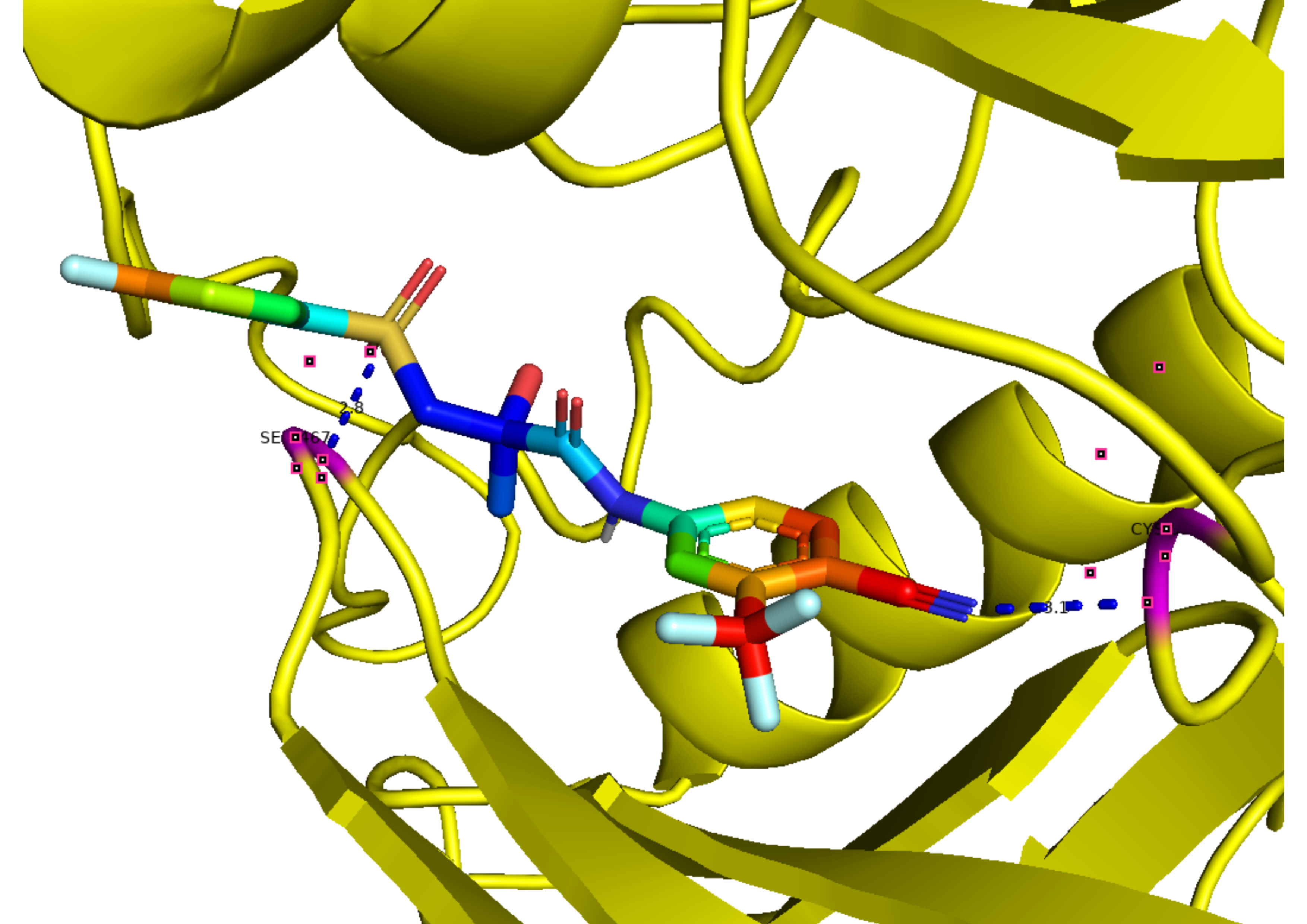


B2


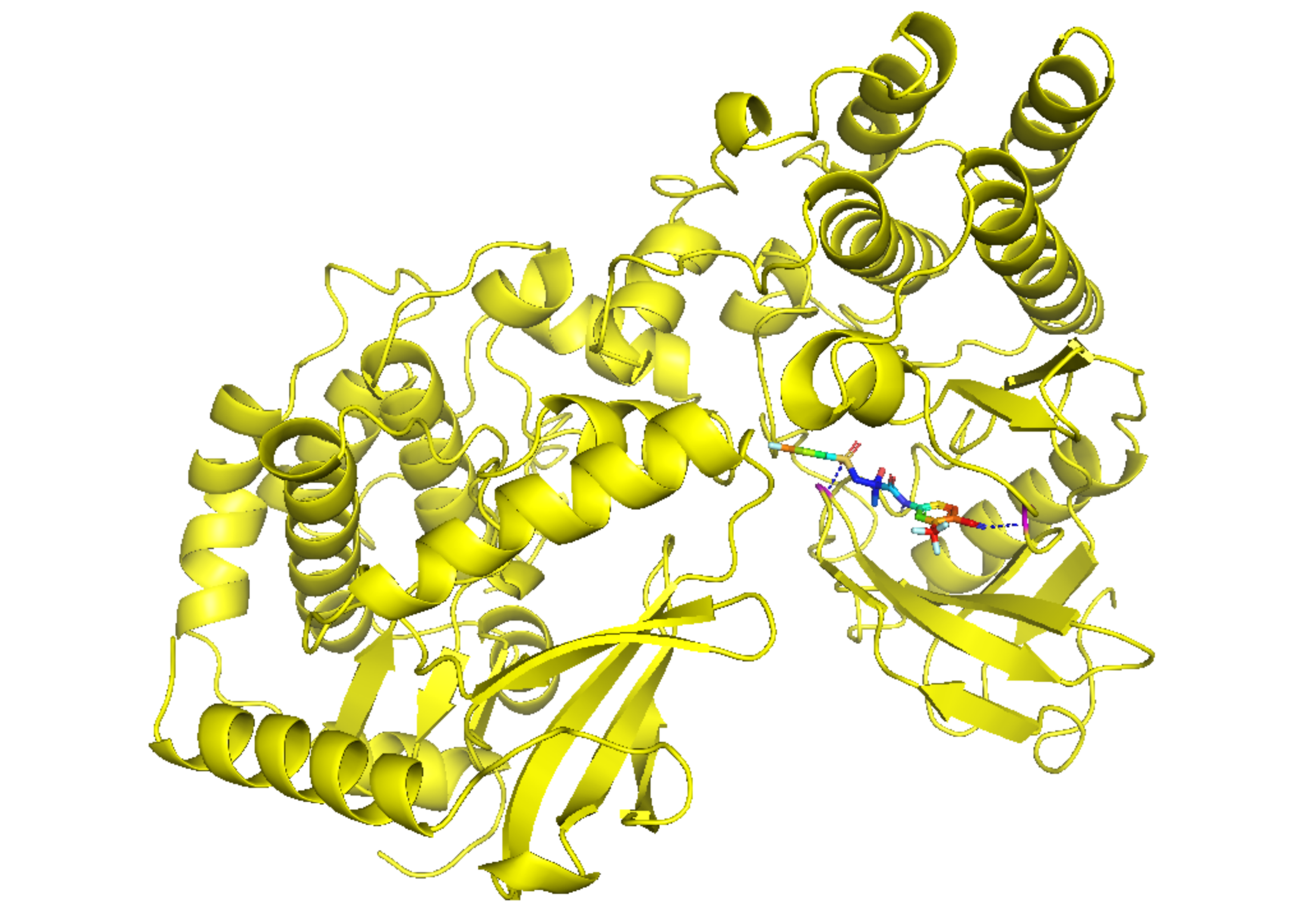


C1


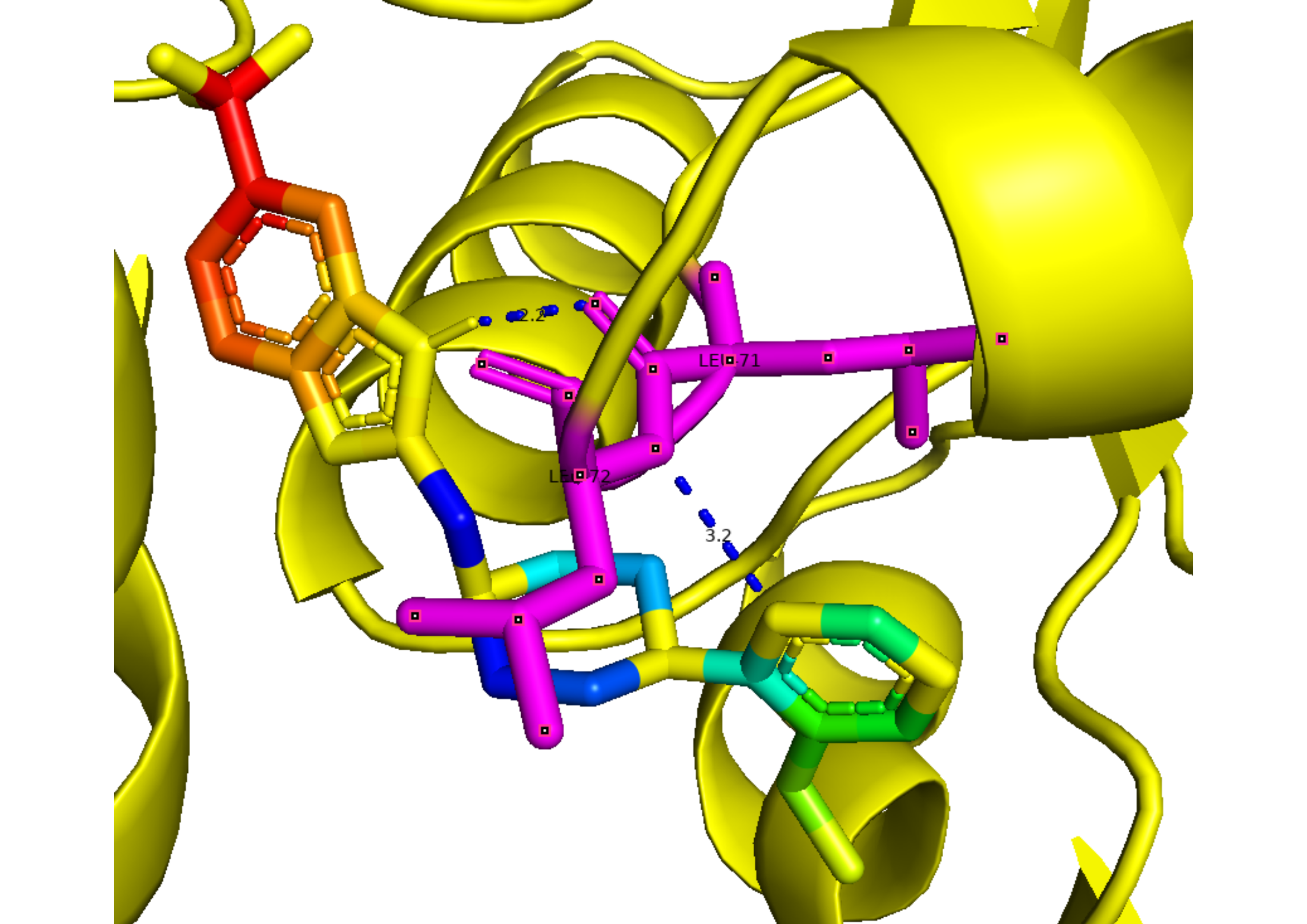


C2


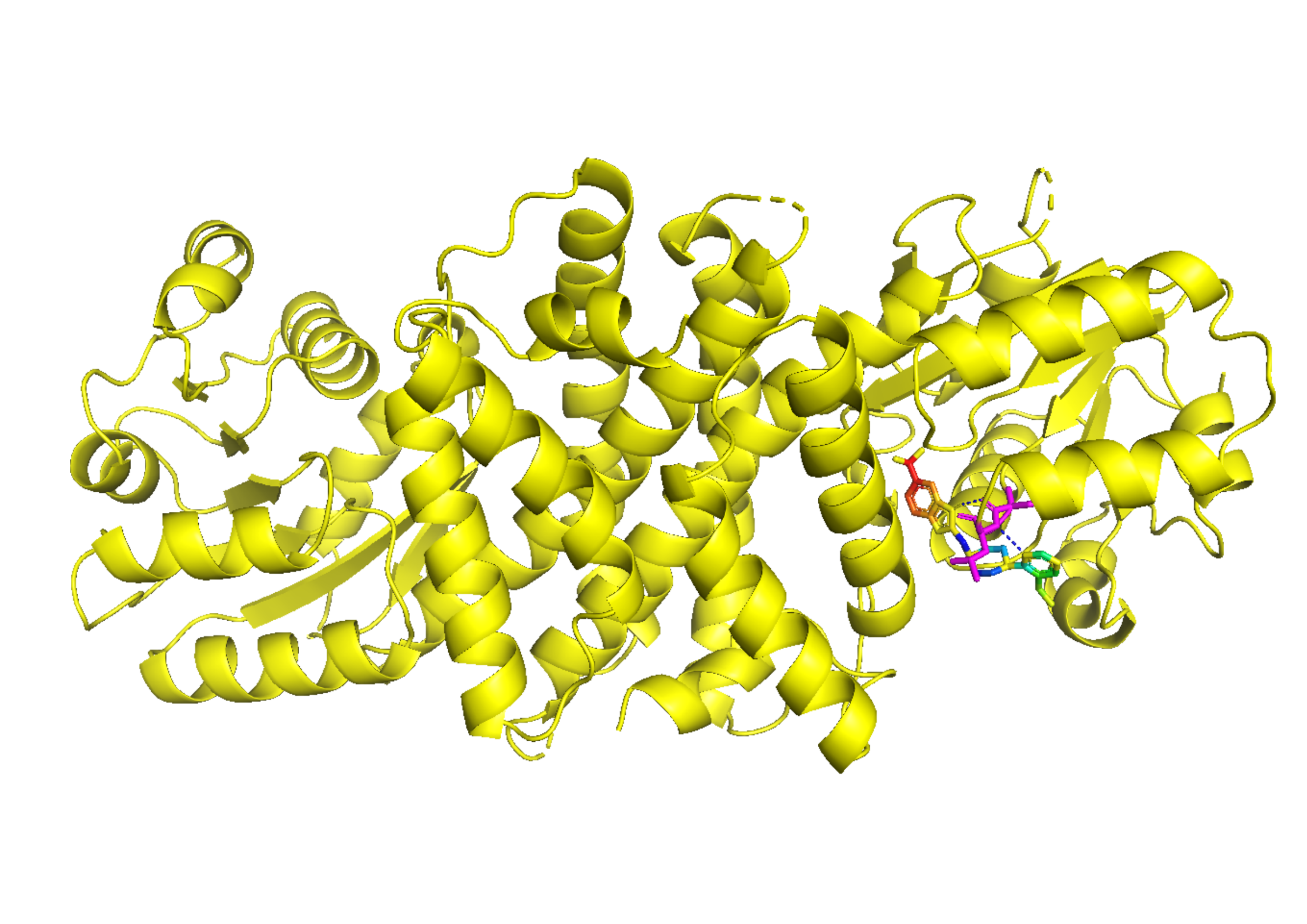
D1


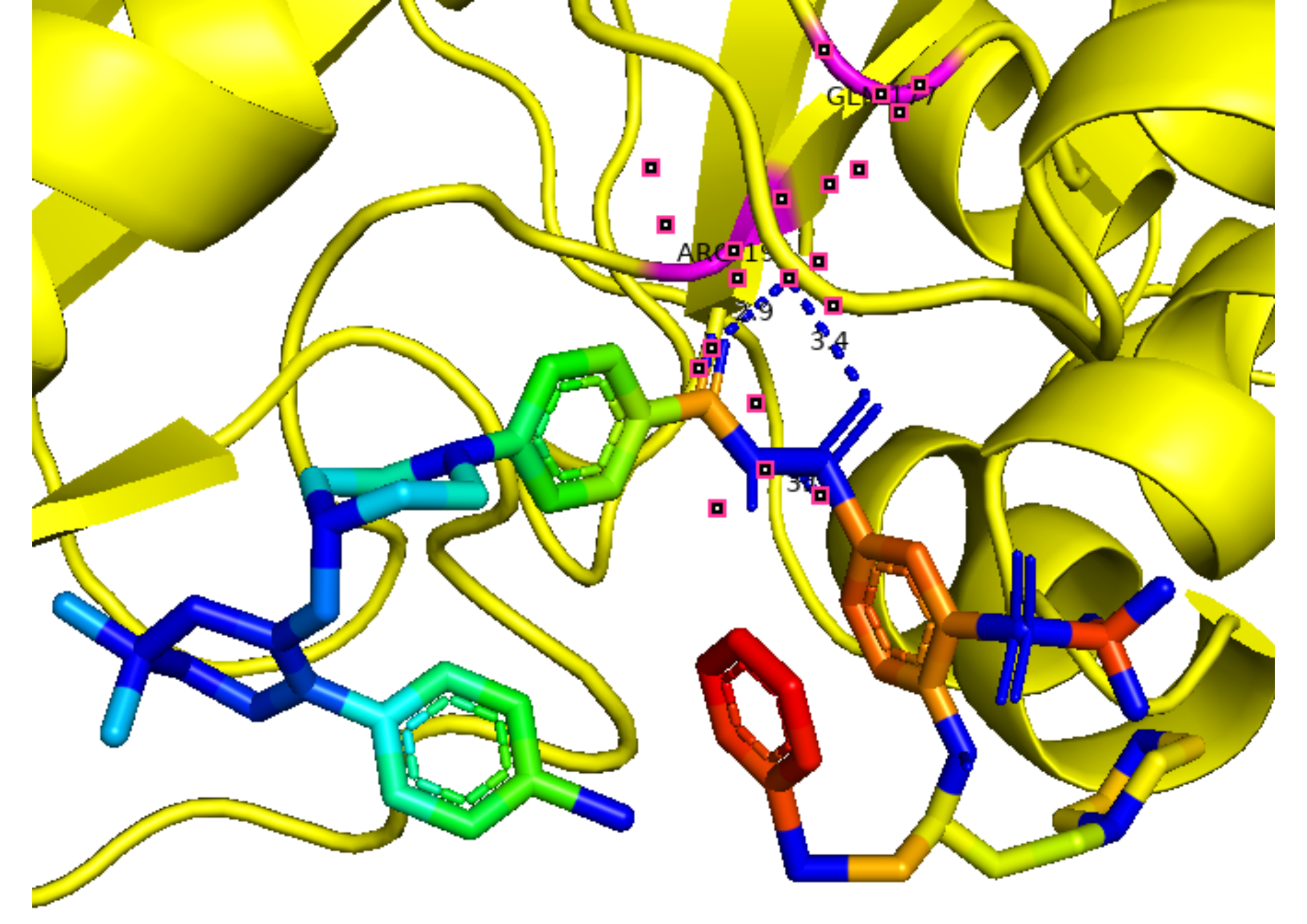


D2


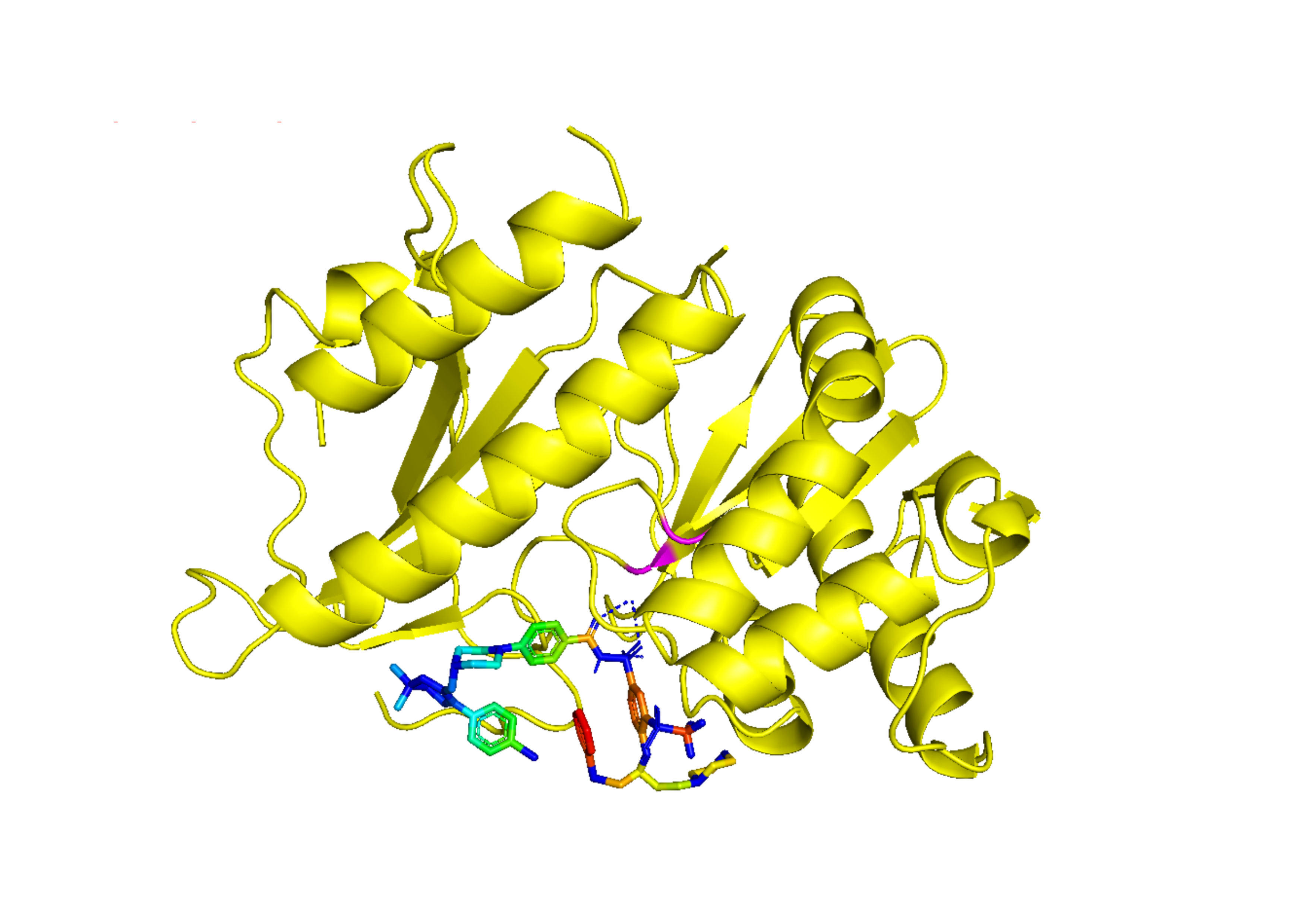


E1


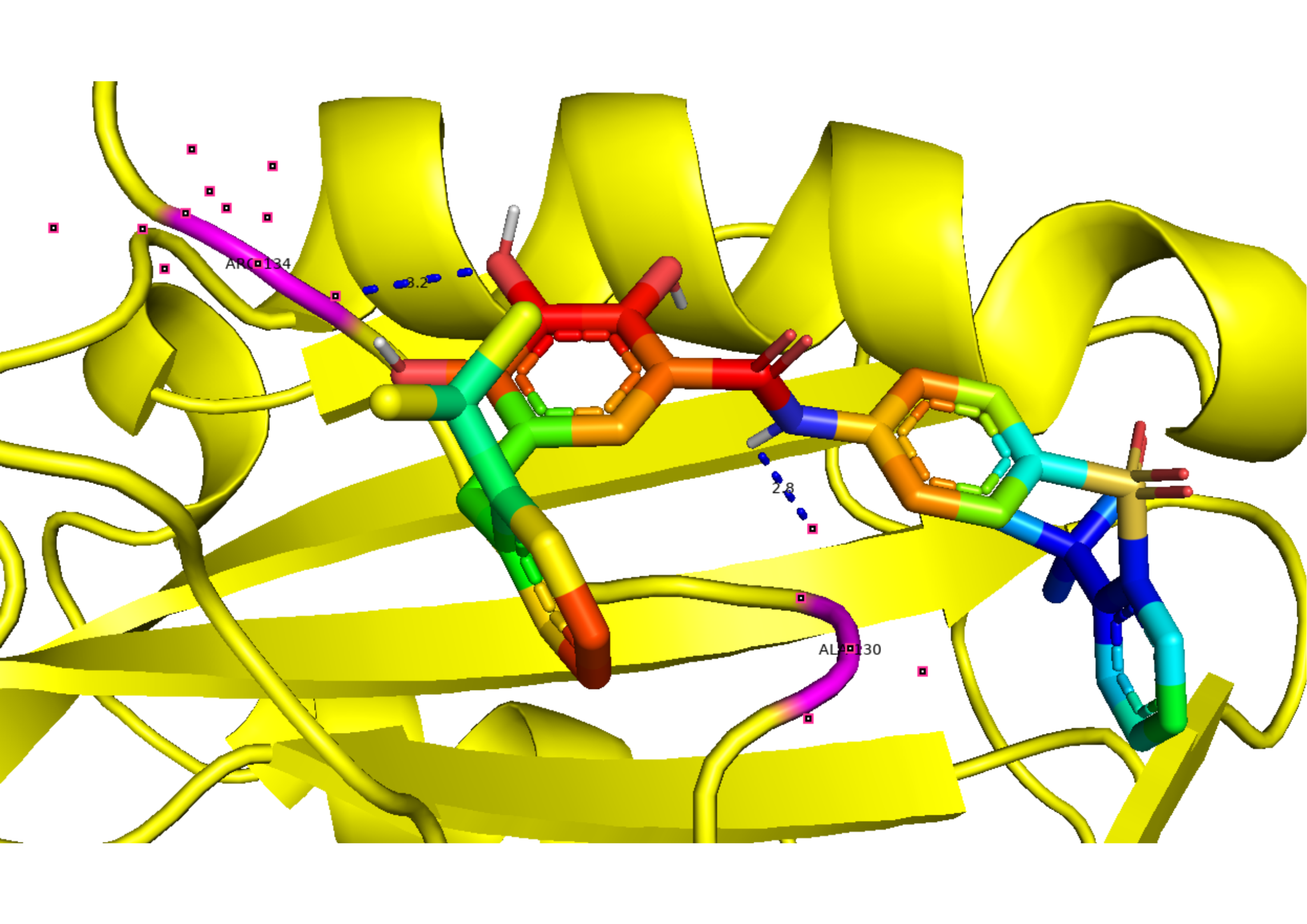


E2


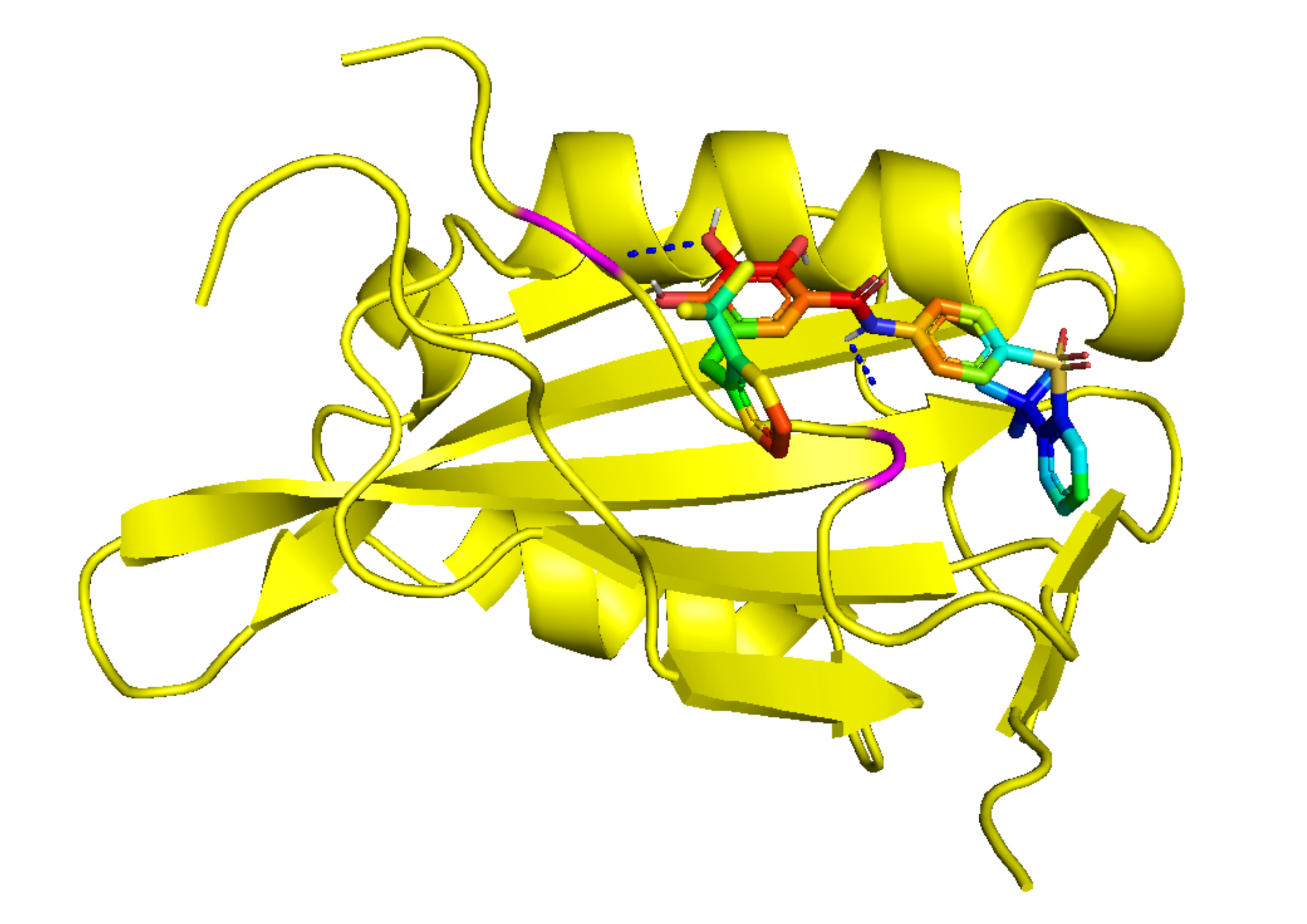

Supplement: Supplementary file 2 — Supplementary Material 2. [file 12672_2026_5118_MOESM2_ESM.docx]

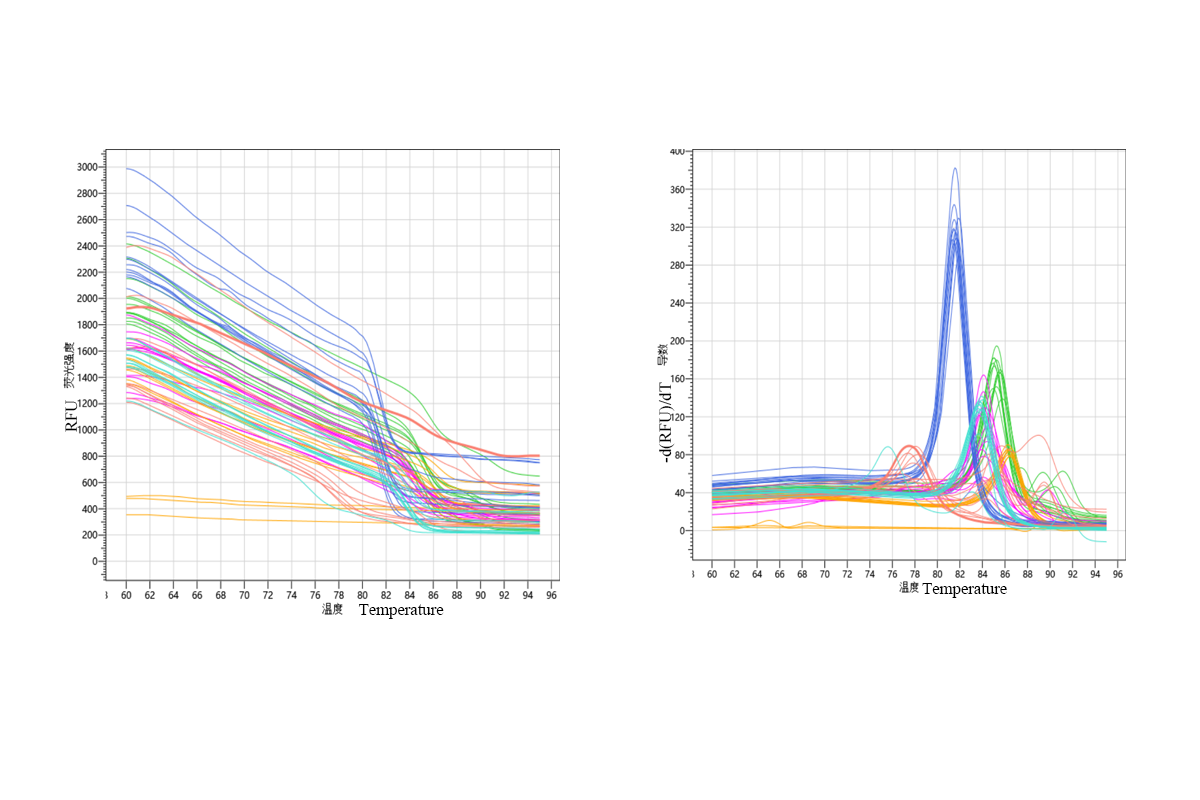

Supplement: Supplementary file 3 — Supplementary Material 3. [file 12672_2026_5118_MOESM3_ESM.png]
